# Gene Expression Signature of Normal Cell-of-Origin Predicts Ovarian Tumor Outcomes

**DOI:** 10.1371/journal.pone.0080314

**Published:** 2013-11-26

**Authors:** Melissa A. Merritt, Stefan Bentink, Matthew Schwede, Marcin P. Iwanicki, John Quackenbush, Terri Woo, Elin S. Agoston, Ferenc Reinhardt, Christopher P. Crum, Ross S. Berkowitz, Samuel C. Mok, Abigail E. Witt, Michelle A. Jones, Bin Wang, Tan A. Ince

**Affiliations:** 1 Department of Biostatistics and Computational Biology, Dana-Farber Cancer Institute, Boston, Massachusetts, United States of America; 2 Division of Gynecologic Oncology, Department of Obstetrics and Gynecology, Brigham and Women’s Hospital, Boston, Massachusetts, United States of America; 3 Department of Epidemiology, Harvard School of Public Health, Boston, Massachusetts, United States of America; 4 Department of Cell Biology, Harvard Medical School, Boston, Massachusetts, United States of America; 5 Division of Women's and Perinatal Pathology, Department of Pathology, Brigham and Women’s Hospital, Boston, Massachusetts, United States of America; 6 Department of Gynecologic Oncology, University of Texas MD Anderson Cancer Center, Houston, Texas, United States of America; 7 Department of Pathology, Interdisciplinary Stem Cell Institute and Braman Family Breast Cancer Institute, Miller School of Medicine, University of Miami, Miami, Florida, United States of America; Cedars-Sinai Medical Center, United States of America

## Abstract

The potential role of the cell-of-origin in determining the tumor phenotype has been raised, but not adequately examined. We hypothesized that distinct cells-of-origin may play a role in determining ovarian tumor phenotype and outcome. Here we describe a new cell culture medium for *in vitro* culture of paired normal human ovarian (OV) and fallopian tube (FT) epithelial cells from donors without cancer. While these cells have been cultured individually for short periods of time, to our knowledge this is the first *long-term* culture of *both* cell types from the *same donors*. Through analysis of the gene expression profiles of the cultured OV/FT cells we identified a normal cell-of-origin gene signature that classified primary ovarian cancers into OV-like and FT-like subgroups; this classification correlated with significant differences in clinical outcomes. The identification of a prognostically significant gene expression signature derived solely from normal untransformed cells is consistent with the hypothesis that the normal cell-of-origin may be a source of ovarian tumor heterogeneity and the associated differences in tumor outcome.

## Introduction

There are marked differences in cellular morphology, mutational spectrum and therapeutic response among the various tumor subtypes that arise from the same organ. A common explanation for these differences has been genetic heterogeneity in the tumors of different patients [1]. Hence, the identification and targeting of patient specific mutations presents an attractive paradigm to guide the design of personalized cancer therapeutics.

An emerging and complementary hypothesis is that phenotypic differences among different subtypes of tumors arising in a single tissue may also be imposed by cell autonomous features unique to the normal cell-of-origin [2], [3]. We and others have shown that transformation of different normal cell subpopulations with identical oncogenes can result in very different tumor behaviors. For example, while some normal breast cell subpopulations gave rise to highly tumorigenic and metastatic adenocarcinomas, other breast cell subpopulations - isolated from the same patients and transformed with identical oncogenes - gave rise to morphologically distinct, weakly tumorigenic and non-metastatic tumors, suggesting that the normal cell-of-origin may be an important factor in determining the associated tumor phenotype [2].

Human ovarian carcinomas are a particularly intriguing group of neoplasms where the normal cell-of-origin may play an important role. The different histopathologic subtypes of epithelial ovarian cancer - serous, endometrioid, clear cell, mucinous, transitional - have been thought to arise in a number of different normal cell types including the ovarian surface epithelium and epithelial lined ovarian inclusion cysts [4]. It also has been suggested that some endometrioid and clear cell ovarian carcinomas may arise from ectopic uterine endometrium (endometriosis) implanted on the ovary [5], [6].

The fallopian tube fimbria epithelium has emerged as an additional candidate cell-of-origin for high grade serous ovarian carcinoma based on findings of morphologically dysplastic areas in normal fallopian tubes from women predisposed to ovarian cancer [7], and the presence of p53 mutations that were identical between these precursor lesions and the matched invasive ovarian carcinomas in the same patients [8], [9]. High grade serous carcinomas are typically associated with p53 mutations [10], [11], and together these observations suggest that the fallopian tube epithelium is a likely cell-of-origin for high grade serous ovarian carcinoma [12].

While the above evidence suggests that ovarian carcinomas may arise from a variety of different cell types, a gene expression signature that could identify the putative cell-of-origin of a particular type of ovarian cancer has been lacking. Thus, our objective was to identify a gene expression signature that could identify the normal cell-of-origin of ovarian carcinomas. Towards this goal, it was necessary to develop a new cell culture medium and methods to isolate and propagate normal ovarian epithelium and fallopian tube epithelium *as paired cultured cells from the same individuals*.

In the standard culture media that were available (e.g., MCDB 105/Medium 199, DMEM/Ham’s F12 and MEM) it was not possible to culture normal ovarian surface epithelial cells for more than 2–12 population doublings [13], [14], [15] and normal fallopian tube epithelial cells were cultured for up to 10 population doublings [16] or three passages [17]. To our knowledge previous investigators have not tested whether the same media could be used to propagate both ovarian and fallopian tube epithelial cells.

We previously described a novel chemically-defined cell culture medium (WIT) that could support the long-term growth of normal human breast epithelial cells without using undefined components such as serum, feeder-layers, tissue extracts or pharmacological reagents [2]. We tested the WIT medium developed for breast cells and found that it did not support the growth of normal ovarian or fallopian tube cells. In the current study we describe the modification of the WIT medium (WIT-fo) to culture normal ovarian epithelial and fallopian tube epithelial cells. Using the newly developed WIT-fo media and associated cell culture methods, we isolated and cultured paired normal ovarian and fallopian tube epithelial cells from the same individuals, identified a gene signature that distinguished these cell types and used this information to classify primary ovarian tumors as fallopian tube epithelial (FT)-like and ovarian epithelial (OV)-like. The FT/OV-like classification provides data to assess similarities between these normal cells and the different ovarian cancer subtypes and importantly this classification is associated with clinically relevant differences in patient survival.

## Materials and Methods

### Ethics statement

Scrapings from the normal ovary and fallopian tube were collected using a kittner from two donors who were treated at the Brigham and Women’s Hospital for benign gynecologic disease following an institutional review board approved protocol to collect discarded tissues that waived the need for consent (Supplementary Methods in File S1). The cells described in this study are primary cell cultures established directly from tissue samples collected during the course of the study.

The protocol for tumorigenesis experiments in immunocompromised mice was approved by the Harvard Standing Committee on Animals (#04639). All experiments were performed in compliance with relevant institutional and national guidelines and regulations.

### Culture of normal human fallopian tube and ovarian epithelium

The most successful method for cell culture establishment was to immediately place the fallopian tube and ovarian cells in WIT-fo culture media and transfer the cells to a tissue culture flask with a modified surface treatment (Primaria, BD, Bedford, MA) and incubate at 37°C with 5% CO_2_ in ambient air. We strongly recommend the use of Primaria culture plates since it was nearly impossible to grow these cells using standard tissue culture plastic ware.

WIT medium was previously described [2] (Stemgent, Cambridge, MA) and WIT-fo is a modified version of this medium optimized for fallopian tube and ovarian epithelial cells. To prepare WIT-fo medium, the WIT medium was supplemented with EGF (0.01 ug/mL, E9644, Sigma-Aldrich, St. Louis, MO), Insulin (20 ug/mL, I0516, Sigma-Aldrich), Hydrocortisone (0.5 ug/mL, H0888, Sigma-Aldrich), Cholera Toxin (25ng/mL, 227035, Calbiochem, EMD Millipore, Billerica, MA) and low concentrations (0.5 – 1%) of heat inactivated fetal bovine serum (HyClone, Thermo Fisher Scientific, Waltham, MA) (Supplementary Methods in File S1).

After 10–15 days, during which the medium was changed every 2–3 days, cells were lifted using 0.05% trypsin at room temperature (∼15 seconds exposure), then trypsin was inactivated in 10% serum-containing medium, followed by centrifugation of cells in polypropylene tubes (500×g, 4 minutes) to remove excess trypsin and serum. Subcultures were established by seeding cells at a minimum density of 1×10^4^/cm^2^. Cell culture medium was replaced 24 hrs after re-plating cells and every 48–72 hours thereafter.

We tested several previously described media formulations to culture ovarian and fallopian tube epithelial cells [18], [19], [20], [21], [22], [23], however, none of these media supported the long-term propagation of normal ovarian or fallopian tube epithelium (Supplementary Methods in File S1). Cell immortalization and transformation of the normal cells with defined genetic elements (following protocols approved by the Committee on Microbiological Safety) was carried out as previously described [2] (Supplementary Methods in File S1). The FNE, OCE, FNLER and OCLER cells described in this manuscript will be available from the Ince laboratory upon request. Experimental conditions for western blotting, live cell imaging and FACS are described in the Supplementary Methods in File S1.

### Microarray analysis

Total RNA was extracted from cells that were cultured in normal WIT-fo media conditions using the RNeasy Mini kit (Qiagen, Valencia, CA) and hybridized onto Affymetrix HG U133 Plus 2.0 microarrays (Affymetrix Inc., Santa Clara, CA) (Supplementary Methods in File S1). The FNE versus OCE signature was compared to two publicly available ovarian cancer datasets that were generated using similar methods in order to minimize microarray platform and methodological bias; datasets generated using total unamplified RNA, isolated from fresh frozen ovarian cancers and profiled using Affymetrix microarray platforms were selected. Microarrays of four hTERT immortalized cell lines (OCE, FNE) from two patients (Gene Expression Omnibus (GEO) GSE37648) as well as publicly available ovarian cancer gene expression datasets by Wu *et al*. [24] (GSE6008) and Tothill *et al*. [25] (GSE9891) were independently normalized using vsnrma [26]. To identify genes that were differentially expressed between paired hTERT immortalized FNE versus OCE, we applied a modified t-test (False Discovery Rate [FDR] [27] adjusted *P*<0.05) using the duplicate correlation function in Limma [28] to account for differences between patients (Supplementary Methods in File S1).

To classify human ovarian tumors as FT-like and OV-like, we selected the most highly significant ten probesets with unique gene symbols that were over-expressed in either FNE or OCE. We then calculated the sum of the normalized expression values of these ten probesets in the ovarian cancer gene expression datasets by weighting FNE genes by (+1) and OCE genes by (–1) resulting in an overall signature expression score for each tumor (i.e., a tumor with a higher score is more FT-like). We then fit a bimodal distribution of Gaussian curves to this score using mixture modeling [29] to classify ovarian tumors as FT-like or OV-like (Supplementary Methods in File S1).

The clinical characteristics of patient tumors that were classified as FT-like or OV-like were compared using ordinal logistic regression (grade, stage) and Fisher’s Exact Test (histologic subtype). To assess whether the FT-like/OV-like classification was associated with differences in patient survival, univariate *P*-values associated with Kaplan-Meier plots were calculated using the log-rank test and multivariate *P*-values (adjusting for tumor grade and stage, serous histologic subtype, patient age and the presence of residual disease) were calculated using the Cox proportional hazards test. Use the following link (http://compbio.dfci.harvard.edu/pubs/OCFNLER/OCFNLER.zip) to download the R script and associated datasets to replicate the microarray analyses. Analyses were conducted using R (v2.10.1).

### Analysis of tumorigenicity and metastasis

The analysis of tumorigenicity was carried out as previously described [2]. Briefly, single-cell suspensions were prepared in a Matrigel: WIT-fo mixture (1:1) and 1 million cells per 100 µl volume were injected in one intraperitoneal (IP) and two subcutaneous (SC) sites per mouse. Tumor cell injections were performed on 6–8 week old female immunodeficient nude (Nu/Nu) mice (Charles River Laboratories International, Inc, Wilmington, MA). Mice were euthanized by CO_2_ asphyxiation followed by cervical dislocation. Tumors were harvested 5 to 9 weeks after implantation of tumorigenic cells from tissue culture into IP and SC sites in nude mice.

## Results

### Establishment of normal primary ovarian and fallopian tube epithelial cultures

**Figure 1:**
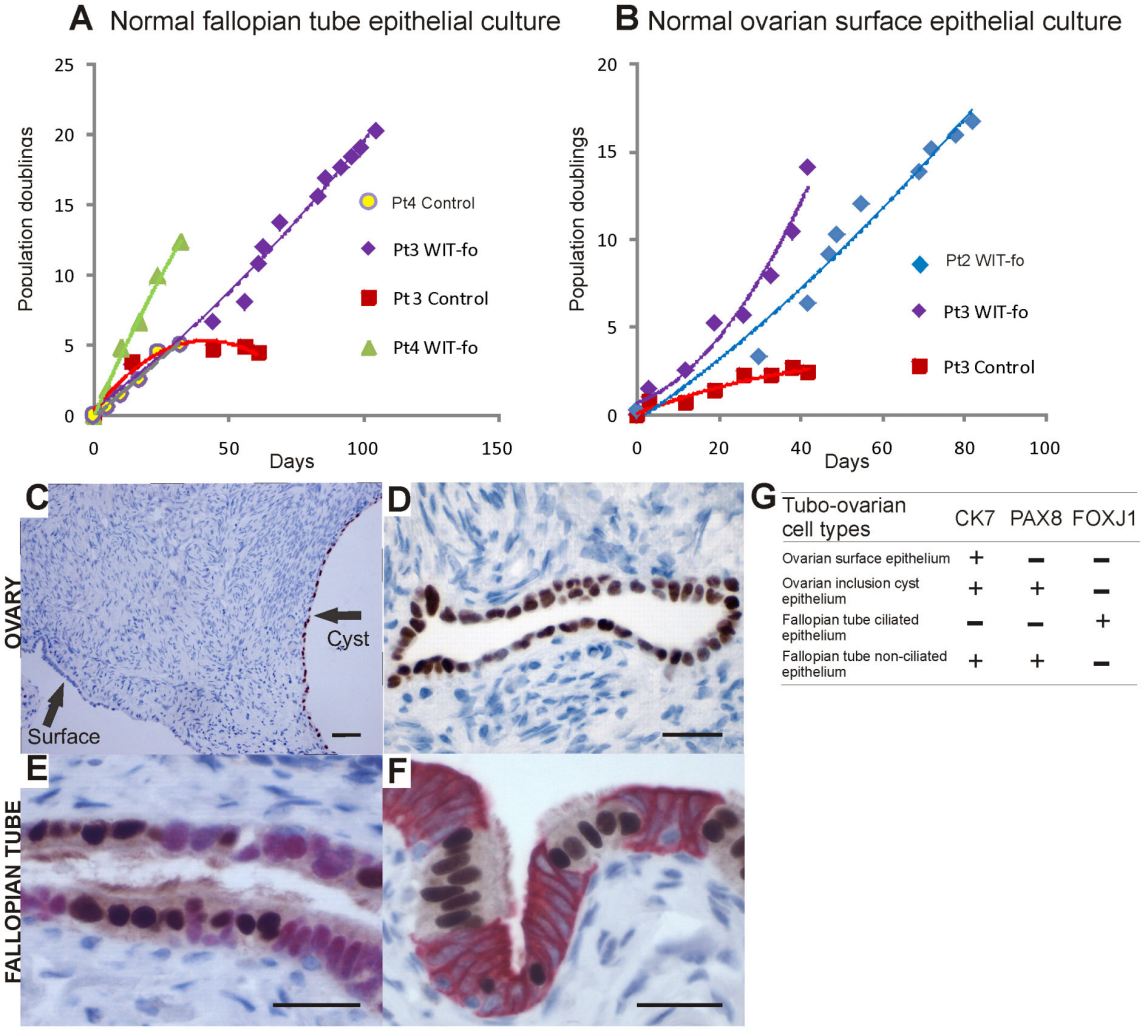
Culture and characterization of normal ovarian epithelial and fallopian tube epithelial cells. **A**, Replicate plates of normal fallopian tube epithelial cells were cultured in WIT-fo medium (green and purple lines) or standard medium (red, grey lines, 1:1 mixture of Dulbecco’s Modified Eagle’s Medium (DMEM) and Ham’s F12, supplemented with 0.1% BSA, 5% serum). In WIT-fo medium, normal fallopian tube epithelial cells from two different patients divided continuously for at least 30 days and > 100 days and reached at least 12 and >20 population doublings respectively (green and purple lines). In contrast, matched cells from the same donors growth arrested in the DMEM/Ham’s F12 medium (red, grey lines). Normal fallopian tube epithelial cells were isolated from additional patients 3 and 4. **B**, Normal ovarian epithelial cells were cultured in WIT-fo medium (purple and blue lines) or standard medium (red line, MCDB 105/Medium 199 (M199) (1∶1 mixture) with 10% FBS and 2 mm l-glutamine). Primary normal ovarian epithelial cells were from patients 2 and 3 (cells cultured in standard medium were from patient 3). Matched cells from patient 3 growth arrested in the MCDB105/M199 medium (red line). **C-D**, Normal human ovarian tissue; immunoperoxidase staining of formalin-fixed paraffin embedded (FFPE) sections with PAX8 demonstrates that ovarian inclusion cyst epithelium is PAX8^+^ (brown nuclear stain) (**C-D**) while ovarian surface epithelium is in general PAX8 negative (**C**), rare presence of rare Pax8 positive cells have been reposted on the ovarian surface. **E,** Normal human fallopian tube tissue; double immunoperoxidase staining of FFPE sections shows ciliated cells are FOXJ1^+^ (nuclear brown) and non-ciliated cells are PAX8^+^ (nuclear red). **F,** Normal human fallopian tube tissue; double immunoperoxidase staining of FFPE sections shows that ciliated cells are FOXJ1^+^ (nuclear brown) and non-ciliated cells are CK7^+^ (cytoplasmic red) (scale bar (C-F)  =  20 µM). **G,** Summary of cell type specific characterization markers.

We isolated paired normal ovarian and fallopian tube epithelial cells collected from separate scrapings of the ovarian surface and the fimbriated end of the fallopian tube using an endoscopic kittner (Supplementary Methods in File S1) from two postmenopausal patients following an IRB approved protocol to collect discarded tissues. Both patients were cancer-free and underwent surgery for benign gynecologic conditions at the Brigham and Women’s Hospital. In order to culture these cells, we modified the chemically-defined WIT medium that we previously described [2]; hereafter the culture medium optimized for normal fallopian tube and ovarian epithelial cells is referred to as WIT-fo. We compared the long-term growth of fallopian tube and ovarian epithelial cells in WIT-fo medium with standard media that have been previously used to culture ovarian epithelium and fallopian tube epithelium [19], [22] by plating cells from the same donor in replicate plates using WIT-fo or standard media (Fig. 1A and B).

It was possible to propagate normal ovarian epithelium and fallopian tube epithelium in WIT-fo medium beyond 10 population doublings, which corresponds to an >1000-fold net increase in cell numbers. In contrast, ovarian and fallopian tube epithelium could not be propagated in standard cell culture media beyond a few population doublings. Hence, we were not able to establish long-term cultures of normal ovarian epithelium or fallopian tube epithelium in any of the previously described media, including the unmodified WIT medium that was originally developed to culture normal breast epithelial cells [2] (Supplementary Methods in File S1).

### Establishment and characterization of immortalized ovarian and fallopian tube cultures

Immortalization of normal human ovarian epithelial cultures has been previously attempted using viral oncogenes such as HPV E6/E7 and SV40 T/t [30], [31]; however, this method also increases genomic instability and can cause the accumulation of DNA mutations that could significantly alter the gene expression profiles in the immortalized cells as compared with their finite lifespan counterparts. Furthermore, these SV40 T/t and E6/E7 transformed cells are not immortal because the genetic instability eventually results in crisis and cell death within weeks to several months of continuous culture [32].

**Figure 2:**
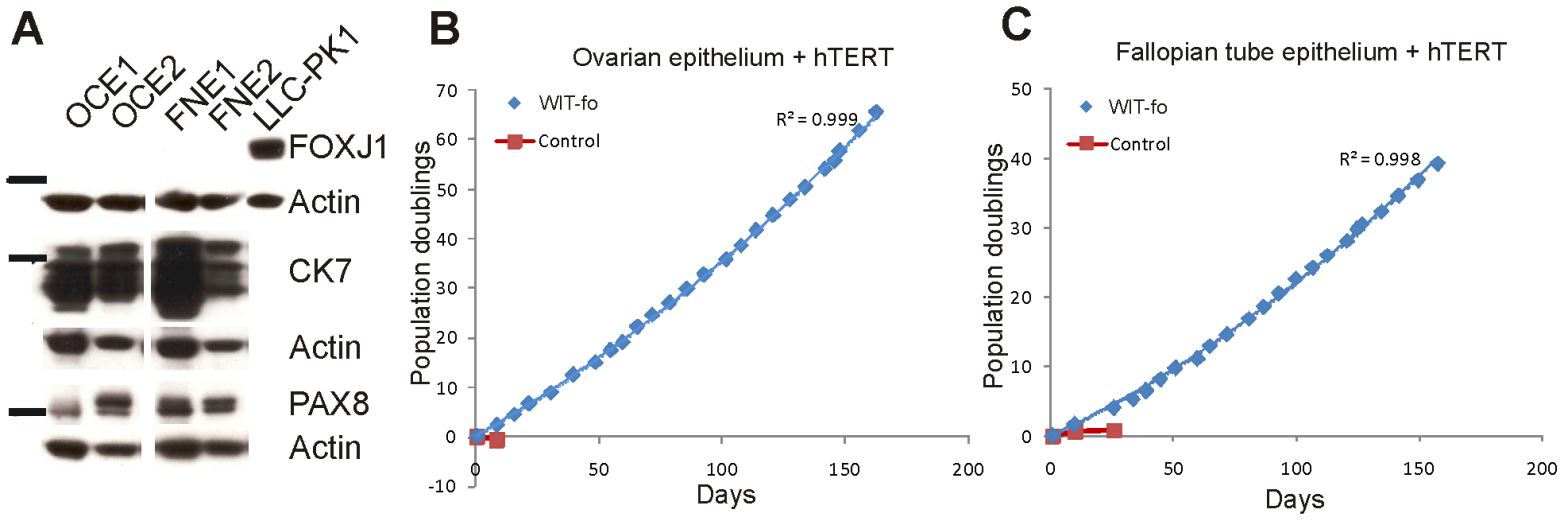
Immortalization of ovarian and fallopian tube epithelial cells with hTERT. **A**, Immunoblotting of whole cell extracts demonstrates that hTERT immortalized ovarian epithelium (OCE) and fallopian tube non-ciliated epithelium (FNE) were CK7^+^/PAX8^+^/FOXJ1¯. Pig kidney cells (LLC-PK1) were included as positive controls for FOXJ1. Each bar marks 52 kDa. **B,** OCE cells from patient 1 were cultured in WIT-fo medium (blue line) or standard medium (red line, MCDB 105/Medium 199 (M199) (1∶1 mixture) with 10% fetal bovine serum and 2 mm l-glutamine). In WIT-fo medium OCE cells divided continuously for >150 days and reached 70 population doublings (blue line) while matched cells from the same donor growth arrested in the control medium (red line). **C**, FNE cells from patient 1 were cultured in WIT-fo medium (blue line) or in standard medium (red line, 1∶1 mixture of Dulbecco’s Modified Eagle’s Medium (DMEM) and Ham’s F12, supplemented with 0.1% BSA and 5% serum). In WIT-fo medium FNE cells divided continuously for >150 days and reached 40 population doublings (blue line). In contrast, matched cells from the same donor growth arrested in the standard medium (red line). R^2^ values indicate the fit of a polynomial line to the data points.

As an alternative to viral oncogenes, we and others have shown that ectopic hTERT expression can be used to immortalize human cells without causing genomic instability [2], [33]. We introduced hTERT into the normal ovarian surface and fallopian tube epithelial cells to create immortalized derivatives, hereafter referred to as **OCE** (ovarian epithelium) and **FNE** (fallopian tube non-ciliated epithelium). In order to characterize OCE and FNE cells, we compared their phenotype with the staining patterns of ovarian and fallopian tube cells in normal human tissues. Several proteins including PAX8, FOXJ1 and CK7 are expressed in a cell type specific manner in the normal human ovary and fallopian tube [21], [34], [35]. CK7 is expressed in ovarian surface and inclusion cyst epithelium as well as in all fallopian tube epithelium, and was used to confirm the epithelial nature of the cultured cells. Many ovarian inclusion cysts are entirely lined by PAX8^+^ expressing epithelial cells while ∼5% of ovarian surface epithelial cells are PAX8^+^ [35]. In contrast, both cell types are FOXJ1 negative (Fig. 1C and D). In the fallopian tube the two major subpopulations of epithelial cells (non-ciliated and ciliated) also can be distinguished using these markers; nearly all non-ciliated cells are PAX8^+^/FOXJ1¯ while the ciliated cells are PAX8¯/FOXJ1^+^ (Fig. 1E-G). Immunoblotting and immunofluorescence staining of the OCE and FNE cells showed that the staining profiles of the ovarian cells were consistent with the ovarian surface/inclusion cyst epithelium while the fallopian tube epithelial cells were similar to non-ciliated epithelium (Fig. 2A and Fig. S1). Similarities between staining profiles in the cultured OCE/FNE cells and ovarian and fallopian tube cells in human tissues suggest that these staining patterns were not an artifact of the culture conditions.

The morphology of the primary and hTERT immortalized OCE and FNE cells in 2D culture appeared compact and cobblestone-like and/or ‘pseudo-spindly’ depending on the confluence of the culture and other factors such as the time since the cells were split (Fig. S2A and S2B). We did not observe cilia in the FNE cells. The immortalized OCE and FNE cells were cultured continuously beyond 40 population doublings, which corresponds to a 10^12^-fold net increase in cell numbers (Fig. 2B and 2C, blue lines). In contrast, replicate plates of the same cells cultured in standard media (Supplemental Methods in File S1) or when transferred to unmodified WIT medium ceased growing after a few passages (Fig. 2B and 2C, red lines; Supplementary Fig. S3 in File S1).

### Application of a cell-of-origin (ovary versus fallopian tube) gene signature to classify patient ovarian cancers

In order to investigate the cell-of-origin of human epithelial ovarian carcinomas we compared the gene expression profiles between FNE and OCE cells isolated from the same patients, and found that 632 and 525 probesets were significantly up-regulated in FNE or OCE cells, respectively (False Discovery Rate (FDR) adjusted *P*<0.05, Tables S1 and S2). From these gene lists we selected the ten most highly significant differentially expressed probesets with unique gene symbols that discriminated between FNE and OCE cells; five of the probesets were overexpressed in FNE cells (gene symbols: *DOK5*, *CD47*, *HS6ST3*, *DPP6*, *OSBPL3*) and five probesets were overexpressed in OCE cells (*STC2*, *SFRP1*, *SLC35F3*, *SHMT2*, *TMEM164*).

**Figure 3:**
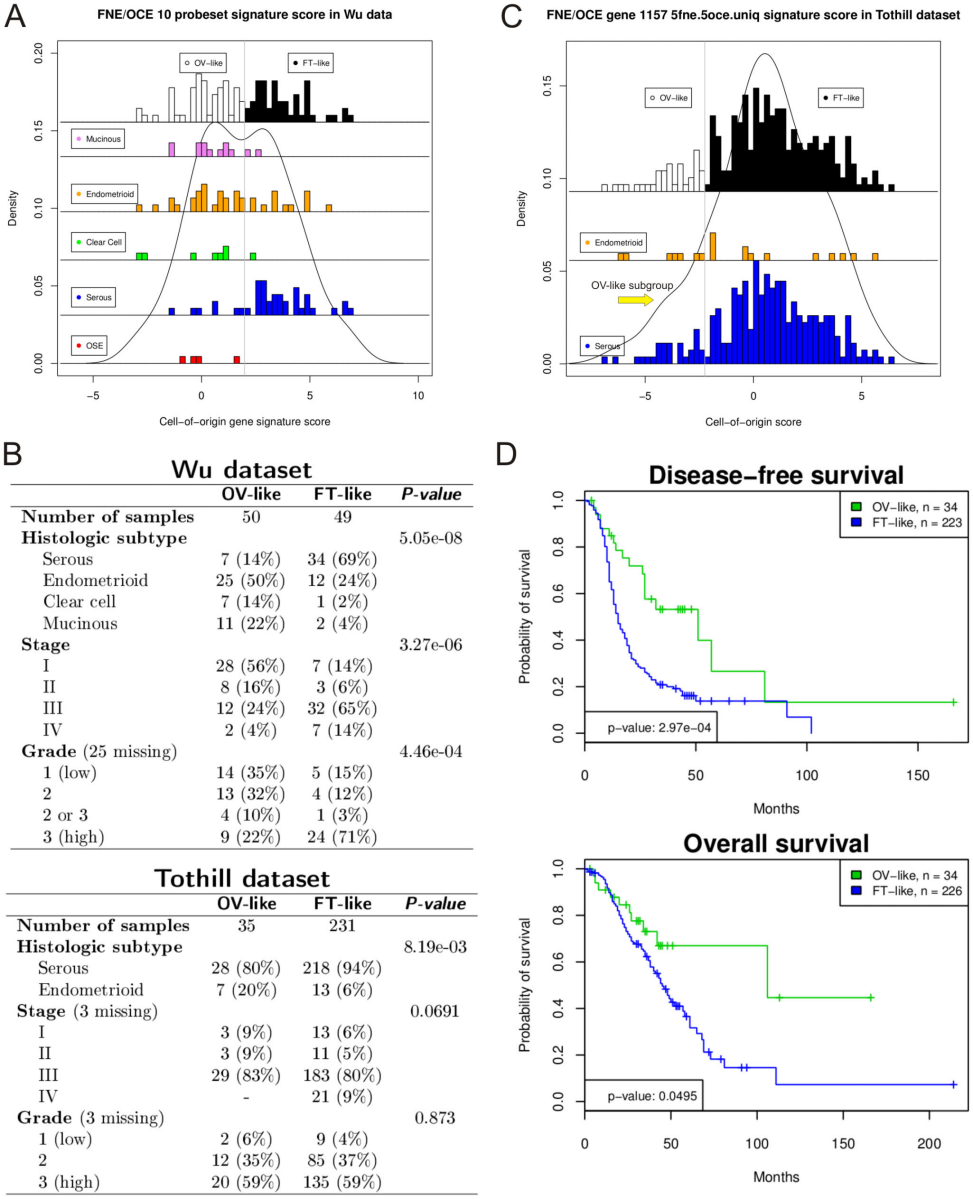
Validation of FNE versus OCE cell-of-origin gene signature in two independent ovarian cancer datasets. **A**, Density plot shows a bimodal distribution of signature scores for 99 ovarian tumor samples and four normal OSE scrapings from the Wu *et al*. dataset. Histograms demonstrate the classification of these samples into normal ovarian epithelial like (OV-like) and normal fallopian tube epithelial like (FT-like) subgroups (top pane), and show the range of signature scores by tumor histological subtype (lower panes). **B**, Association of the OV-like/FT-like tumor classification in the Wu and Tothill datasets with clinical characteristics (*P*-values from logistic regression (grade, stage as ordinal variables) and Fisher’s Exact test (histological subtype)). **C**, Density plot demonstrates a slight left skewing of the signature scores in the Tothill *et al*. dataset which suggests a small subpopulation (arrow) of OV-like tumors. Histograms demonstrate the distribution of signature scores in endometrioid and serous tumors. **D**, Kaplan-Meier plots demonstrate significant differences in the disease-free survival and overall survival between OV-like and FT-like subgroups in the Tothill dataset (univariate *P*-values from the log-rank test are displayed).

In order to validate the FNE versus OCE normal cell-of-origin signature we examined it in previously published gene expression datasets that had profiled normal human fallopian tube epithelium and normal ovarian surface epithelium [36], [37], [38], [39]. It is worth noting that each of these studies examined only one cell type at a time - either ovarian or fallopian tube epithelial cells. Hence, the ovarian and fallopian tube specific expression profiles were from separate studies in which normal ovarian or fallopian tube epithelial cells were isolated from different patients using various collection methods. Despite these differences, the FNE/OCE signature correctly classified all of the microdissected fallopian tube epithelium samples as fallopian tube (FT)-like and all of the cultured ovarian surface epithelial cells as ovary (OV)-like in two different datasets (Fig. S4A and S4B in File S1). Four uncultured normal ovarian surface epithelial brushings in the Wu *et al*. [24] dataset also were correctly classified as OV-like (Fig. 3A). These results indicated that the FNE/OCE normal cell-of-origin signature could be applied across other data sets successfully.

We hypothesized that a residual cell-of-origin signature may be retained in ovarian tumors which could be used to identify their origin retrospectively. Hence, we used the FNE/OCE normal cell-of-origin signature to classify 99 ovarian carcinomas in the Wu *et al*. [24] dataset that included a representation of different histologic subtypes and tumor grades. Due to array platform differences 8/10 probesets were available for this analysis. In this dataset the gene signature scores visualized in a density plot showed a bimodal distribution which supports our binary classification of ovarian tumors into normal FT-like and normal OV-like subgroups (Fig. 3A). Comparisons between cultured cells and tumor tissue gene expression profiles are difficult to carry out for several reasons including the presence of stromal cells in the tumor tissue which may influence the gene expression profile. Fortunately, the tumors in the Wu dataset were microdissected which permits a more direct comparison of gene expression signatures between cultured OCE/FNE cells and ovarian tumor cells.

Consistent with previous studies that suggested that high grade serous cancers might arise in the fallopian tube, we observed that the tumors that were classified as FT-like (similar to the normal FNE cells) in the Wu dataset were of significantly higher stage, higher grade and were predominantly composed of serous adenocarcinomas (*P*≤0.0004 for all comparisons) (Fig. 3B). In contrast, OV-like tumors included predominantly non-serous histologic subtypes and lower grade cancers. Thus, the normal cell-of-origin gene expression signature appeared to split human ovarian tumors in the Wu dataset into two distinct OV-like and FT-like groups with different clinical and histopathological characteristics.

The inclusion of additional probesets (e.g., 20 most highly significant probesets in the FNE versus OCE comparison) to calculate the signature score did not substantially alter the results (data not shown) and is consistent with studies that have shown that the inclusion of additional genes in a signature does not always improve the robustness of the classification [40], [41], [42]. Furthermore, using a different method to evaluate the continuous cell-of-origin signature scores showed similar results; the continuous scores differed significantly between tumor histologic subtypes, grade and stage (*P*≤0.005 for all comparisons, Welch t-test) (Fig. S5A in File S1). We compared these results with randomly permuted ten probeset signatures (Fig. S5B in File S1) and none of the randomly permuted signatures were associated with tumor histologic subtype, grade or stage.

We examined the FNE/OCE cell-of-origin signature in a second ovarian cancer gene expression dataset (Tothill *et al*.) [25]. In contrast to the Wu dataset, the tumors in the Tothill dataset were not microdissected and only two histologic subtypes were represented (246 serous tumors and 20 endometrioid tumors). Possibly due to these differences, we observed a left skewed distribution rather than a bimodal distribution in the signature scores which is consistent with a small subgroup of OV-like tumors (Fig. 3C). Nonetheless, in the Tothill dataset, the FT-like subgroup contained mostly serous tumors (*P* = 0.008) and there was a suggestive enrichment for advanced stage tumors (*P* = 0.07) (Fig. 3B, bottom panel). There was no association with tumor grade in this dataset (*P* = 0.87) possibly due to the small number of low grade lesions (n = 11). Examination of the continuous cell-of-origin signature scores showed similar but non-significant results; there was a suggestive shift to the right (indicating a higher score/more FT-like) for serous versus endometrioid tumors and for stage III/IV as compared with stage I/II tumors (*P* = 0.09 in both comparisons) and no association with tumor grade (data not shown).

To evaluate whether originating from the ovary or fallopian tube may be associated with differences in patient survival, we compared the clinical outcomes between OV-like and FT-like tumors in the Tothill dataset (survival data was not available in the Wu dataset). We found that patients with FT-like tumors had significantly worse disease-free survival (univariate log-rank *P* = 0.0003) and poorer overall survival (*P* = 0.05) (Fig. 3D). Importantly, after adjusting for tumor grade, stage, serous subtype, patient age and residual disease in multivariate analysis, the OV/FT-like subgroups were associated with significant differences in disease-free survival (Cox proportional hazards *P* = 0.01) but not overall survival.

**Figure 4:**
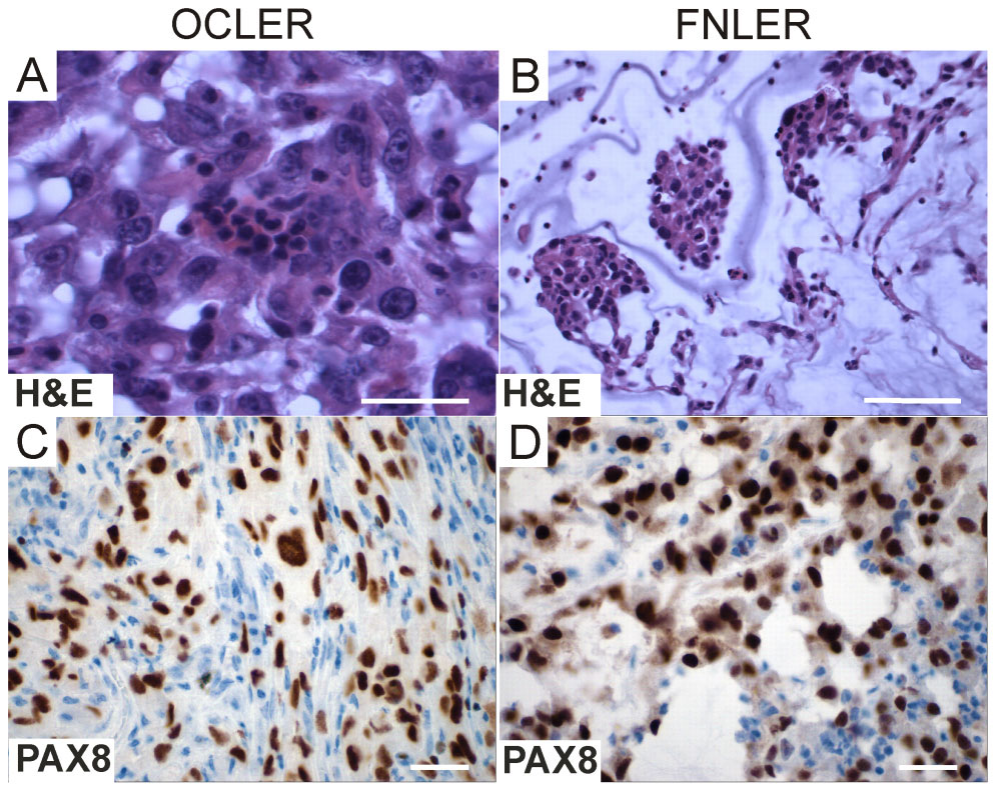
OCLER and FNLER tumor histopathology in immunodeficient nude (Nu/Nu) mice. **A-B**, Hematoxylin and eosin (H&E) stains of representative formalin-fixed paraffin-embedded (FFPE) tumor sections from OCLER (A) and FNLER (B) xenografts revealed focal micropapillary structures. The predominant morphology was diffuse sheets of cells with a poorly differentiated tumor architecture (scale bar  =  20 µM). **C-D**, PAX8 immunoperoxidase stains of representative FFPE tumor sections from OCLER (C) and FNLER (D) xenografts confirmed that xenografts retained their PAX8 expression (scale bar  =  20 µM).

To further test the influence of the normal cell-of-origin on the associated tumor phenotype, we created transformed derivatives of the hTERT immortalized FNE and OCE cells by the sequential introduction of SV40 Large T/small t (SV40T/t) antigen and H-Ras as we described before [2], [43]; these tumorigenic cells are hereafter referred to as FNLER and OCLER, respectively. Equal numbers of transformed FNLER and OCLER cells were injected into the intraperitoneal space and 2× subcutaneous sites of 24 total immunodeficient nude (Nu/Nu) mice (15 and 9 mice were injected with OCLER or FNLER, respectively, at two time points four months apart). Experimental conditions were identical and rates of tumor formation, tumor burden and metastases were similar at both time points and therefore results were combined. Necropsy analyses of mice 5–9 weeks after the tumor cell injection revealed similar rates of xenograft formation, total tumor burden (Table 1) and tumor histopathology (poorly differentiated with focal micropapillary-like architecture) in both cell types (Fig. 4). Both FNLER and OCLER derived tumors were highly invasive into the surrounding intraperitoneal tissues (Fig. S6A and S6B in File S1). Examination of the lungs from mice bearing tumors (tumor mass ≥ 0.5g) revealed striking differences in the propensity to develop lung metastases; FNLER formed metastases in the lungs of 67% of mice (n = 6 mice evaluated) while isogenic OCLER formed metastases in only 13% of the mice (n = 8 mice evaluated) (*P* = 0.04, Mann-Whitney test). A higher number of mice were injected with OCLER cells (versus FNLER) to increase the sample size to investigate lung metastases in OCLER. The metastatic index (number of metastatic cells/total tumor burden) for mouse lungs also was higher in mice bearing FNLER tumors (Fig. S7 in File S1). The presence of the metastatic cells was confirmed in the FFPE lungs with H&E and immunohistochemical staining for p53 (Fig. S6C and S6D in File S1) and SV40 (data not shown). No difference was observed in the total tumor burden or the time of tumor incubation between mice injected with FNLER or OCLER that were examined for lung metastases (Table S3 in File S1). These *in vivo* investigations were exploratory as they were based on a small number of mice; nevertheless, these data, combined with our previous observations that FT-like patient tumors were associated with worse outcome, are consistent with the hypothesis that the normal cell of origin may play a role in determining the associated tumor phenotype.

**Table 1:** Tumor formation, tumor burden and ascites in the OCLER and FNLER xenograft model.

Cell line	Genotype	Tumor formation	Tumor burden a/mouse (g) ± s.d.	Ascites present b
		Number of mice (%)		Number of mice
OCLER	*hTERT*, SV40 *ER*, *HRAS^V12^*	13/15 (87)	3.24 ± 2.08	0/13
FNLER	*hTERT*, SV40 *ER*, *HRAS^V12^*	6/9 (67)	4.94 ± 2.36	3/6
		*P* c = 0.25	*P* c = 0.33	*P* c = 0.007

a Values shown are means ± s.d. across all mice injected with each cell type that had any evaluable tumor mass (the sum of 1× intraperitoneal and 2× subcutaneous sites per mouse).

b Formation of ascites was only evaluated among mice that developed tumors.

c *P*-values were calculated using the Mann-Whitney test.

## Discussion

The possibility that tumor behavior is influenced by the intrinsic characteristics of the normal cell-of-origin in which the cancer-promoting mutations emerge has been raised but has not been extensively studied [2], [44], [45]. Although ovarian cancer has traditionally been thought to arise in the ovarian surface or inclusion cyst epithelium [4] or in endometriosis [5], [6], the more recent discovery of a candidate precursor lesion in the fallopian tube fimbria epithelium suggests that a substantial number of ‘ovarian’ cancers may originate in the fallopian tube [8], [12], [46]. Indeed previous tissue-based studies have reported similarities in gene expression between normal fallopian tube epithelium and papillary serous carcinoma [47], [48]. In the current study we observed that >80% of serous carcinomas in two independent ovarian cancer gene expression datasets were classified as fallopian tube (FT)-like using the FNE versus OCE cell-of-origin signature. This result provides independent support for the hypothesis that a large proportion of high grade serous carcinomas may arise from the non-ciliated fallopian tube epithelium [49].

Although based on small numbers we observed that ≥85% of mucinous and clear cell tumors were classified as ovary (OV)-like, suggesting that these tumors may arise from ovarian epithelium. In contrast, endometrioid adenocarcinomas had a broader spectrum of phenotypes. Based on the cell-of-origin signature in the Wu dataset, 67% of endometrioid cancers were classified as OV-like. In contrast, 65% of endometrioid cancers were in the FT-like subgroup in the Tothill dataset. These observations suggest multiple candidate cells-of-origin for endometrioid tumors.

In the xenograft model we observed that the transformed FNLER and OCLER cells formed undifferentiated tumors with focal micropapillary histology in mice. In previous studies fallopian tube epithelium has been cultured using different standard media conditions and using similar genetic alterations (including *hTERT*, SV40T/t and *HRAS* +/– *C-MYC*); intriguingly, differences in the resulting tumor histopathology have been reported - two of these studies claimed that the histology of xenograft tumors was consistent with human poorly differentiated serous tumors [17], [50] while the third study observed a poorly differentiated mucinous histology [16]. Differences in the resulting tumor phenotype may be due to different strains of mice or different methodologies used to isolate and culture the fallopian tube epithelial cells as we previously described in our breast cancer model [2]. Regardless of the source, these observations suggest that there are different cell subpopulations in the normal fallopian tube that can give rise to tumors with different histologic appearances and tumor behavior. Hence, these findings further support the hypothesis that the normal cell-of-origin is an important source of ovarian tumor heterogeneity.

The FNE versus OCE cell-of-origin signature included probesets that were associated with 10 unique genes, five that are over-expressed in FNE versus OCE (*DOK5*, *CD47*, *HS6ST3*, *DPP6*, *OSBPL3*) and five that are over-expressed in OCE versus FNE (*STC2*, *SFRP1*, *SLC35F3*, *SHMT2*, *TMEM164*). It remains to be determined whether these genes are simply markers of the cell type or they reflect functional differences between these cell types. The ovary has been reported to have high levels of *Stc2* (Stanniocalcin 2) mRNA expression [51] and in a rat model Luo *et al*. [52] suggested that *Stc2* may play a paracrine role in ovarian progesterone biosynthesis. Protein expression of SFRP1, a modulator of Wnt signaling and a stem cell marker, has been reported in normal human ovarian surface epithelium [37] and in fallopian tube fimbria epithelium [53]. CD47 is a cell surface marker that is broadly expressed in normal adult tissues and in human solid tumors including ovarian cancer [54].

In this study we developed a new medium (WIT-fo) and associated methods to culture normal ovarian and fallopian tube cells that were isolated from patients who were cancer-free. The WIT-fo medium described in the current study is distinct from other recently described media formulations which have been used to culture ovarian or fallopian tube cells [16], [17], [55]. For example, Liu *et al*. [55] described the culture of several cell types which included one normal ovarian cell line but no tubal cells; however, no data for population doublings of the ovarian line was provided. This study used ROCK inhibitors to prevent cell death by blocking apoptosis, hence the cell lines derived using this media would be dependent on ROCK inhibitors and on mouse 3T3 fibroblasts as feeder layers. In contrast, we have no such drugs or feeder layers in our cell culture model. Karst *et al*. [17] stated that it was not possible to culture normal fallopian tube cells for >10 passages in their media. Furthermore, when they created immortalized tubal cells by introducing a retroviral vector encoding hTERT they noted that although hTERT alone prevented senescence (≤10 passages) it did not promote cell growth to the extent that allowed for cell line expansion. Lastly, the Shan *et al*. [16] study stated that it was not possible to culture normal tubal cells for >10 population doublings in their medium. These results differ from the culture conditions developed in the current study in which hTERT immortalized OCE and FNE cells were cultured continuously beyond 40 population doublings, which corresponds to a 10^12^-fold net increase in cell numbers.

Recent findings suggest that endometrioid and clear cell ovarian carcinomas may arise from endometriosis implanted on the ovary [5], [6]. Endometriosis was not evaluated in this study because we were not able to develop a cell culture medium for these cells. Endometriosis lesions also are intrinsically different from normal ovarian and fallopian tube epithelial cells because these lesions have activated oncogenic pathways and mutations [12], [56], [57], hence they are not entirely normal. Moreover, the precise normal cell-of-origin for endometriosis is uncertain; it has been postulated that endometriosis may arise due to trans-differentiation of peritoneal mesothelial cells or from uterine endometrium that is ectopically transplanted in the peritoneum [56]. For these reasons, further studies will be needed to evaluate endometriosis as a precursor lesion for ovarian cancer.

Observations in inherited cancer susceptibility syndromes support an important relationship between the cell-of-origin and tumor suppressor genes. For example, although many tumor suppressor genes such as *RB* [58], *BRCA1/*2 [59] or *WT1* [60] are expressed ubiquitously in almost all tissues, their loss often gives rise to tumors specifically in the retina, breast or ovary/fallopian tube and kidney, respectively. These tissue-specific patterns of tumorigenesis may reflect the susceptibility of the particular cell-of-origin to a specific mutation. For example, it was shown that while deletion of *Rb* caused apoptosis in most retinal cell types in mice, it was tolerated in a specific subpopulation of retinal cells which were the cell-of-origin for retinoblastoma [58]. Thus, these cell type specific actions of *Rb* were the mechanism for the tissue specific tumor development in inherited retinoblastoma.

The role of the cell-of-origin in sporadic cancers has been more difficult to elucidate. One reason for this is that by definition the process of carcinogenic transformation destroys the normal cell from which the tumor initiates, which complicates retrospective analyses of the cell-of-origin in tumor tissues. We used an approach that isolates the candidate normal cells-of-origin and compares the gene expression of these cells to those of tumors. While simple as a concept, studies of normal ovarian and fallopian tube epithelial cells have been hampered in part by the lack of a robust cell culture system. To our knowledge the continuous long-term culture of normal fallopian tube epithelium has not been possible using the previously described cell culture media.

In summary, we have described a new culture media and methods that permitted the development of an experimental model of paired hTERT immortalized human ovarian (OCE) and fallopian tube (FNE) epithelial cells from donors who were cancer-free. We observed that patients with FT-like tumors had significantly worse disease-free survival even after adjusting for important prognostic factors such as tumor stage and grade. Notably, the FNE versus OCE signature was derived from normal hTERT immortalized cells that are untransformed and non-tumorigenic. These findings suggest that an intrinsic network of genes expressed by the normal cell-of-origin and retained by the tumor may play an important role in determining the malignant tumor phenotype. These findings suggest that studies of tumor mutations coupled with the knowledge about the cell-of-origin context may be needed to gain a full appreciation of factors leading to differences in tumor behavior.

## Supporting Information

Figure S1 **Immunofluorescence staining of cultured OCE and FNE cells for PAX8 and FOXJ1. A-B**, Immunofluorescence staining shows that OCE and FNE cells are PAX8^+^/FOXJ1¯ while IHOSE cells (immortalized using HPV E6/E7 [Tsao *et al*. 1995, Exp Cell Res 218: 499-507]) were PAX8¯. FNE1 and FNE2 indicate that these cells were derived from patients 1 and 2, respectively. The positive control for FOXJ1 (ciliated pig kidney cells [i.e., LLC-PK1]) all showed positive nuclear staining (data not shown). Experimental conditions for immunofluorescence are detailed in the ‘Antibodies and experimental conditions' table in the Supplementary Methods in File S1.
(TIF)

Figure S2 **Morphology of primary and hTERT-immortalized FNE and OCE cells in WIT-fo medium. A**, Photographs at 10× magnification. **B**, Cropped and enlarged photographs (white frames in Fig. S2A).
(TIF)

Table S1 **Probesets that were up-regulated (n = 632) in comparisons of immortalized fallopian tube non-ciliated epithelium (FNE) versus immortalized ovarian epithelium (OCE) (FDR adjusted** ***P*** **<0.05).**
(PDF)

Table S2 **Probesets that were up-regulated (n = 525) in immortalized ovarian epithelium (OCE) as compared with immortalized fallopian tube non-ciliated epithelium (FNE) cells (FDR adjusted** ***P*** **<0.05).**
(PDF)

File S1 **Combined Supporting Information file including the Supplementary Methods and the specified Supplementary Tables and Figures.**
(DOCX)
