# Supplementary figures and images for: Gene Expression Signature of Normal Cell-of-Origin Predicts Ovarian Tumor Outcomes
Source: PLoS One. 2013 Nov 26;8(11):e80314. doi: 10.1371/journal.pone.0080314 (PMC3841174; doi:10.1371/journal.pone.0080314)

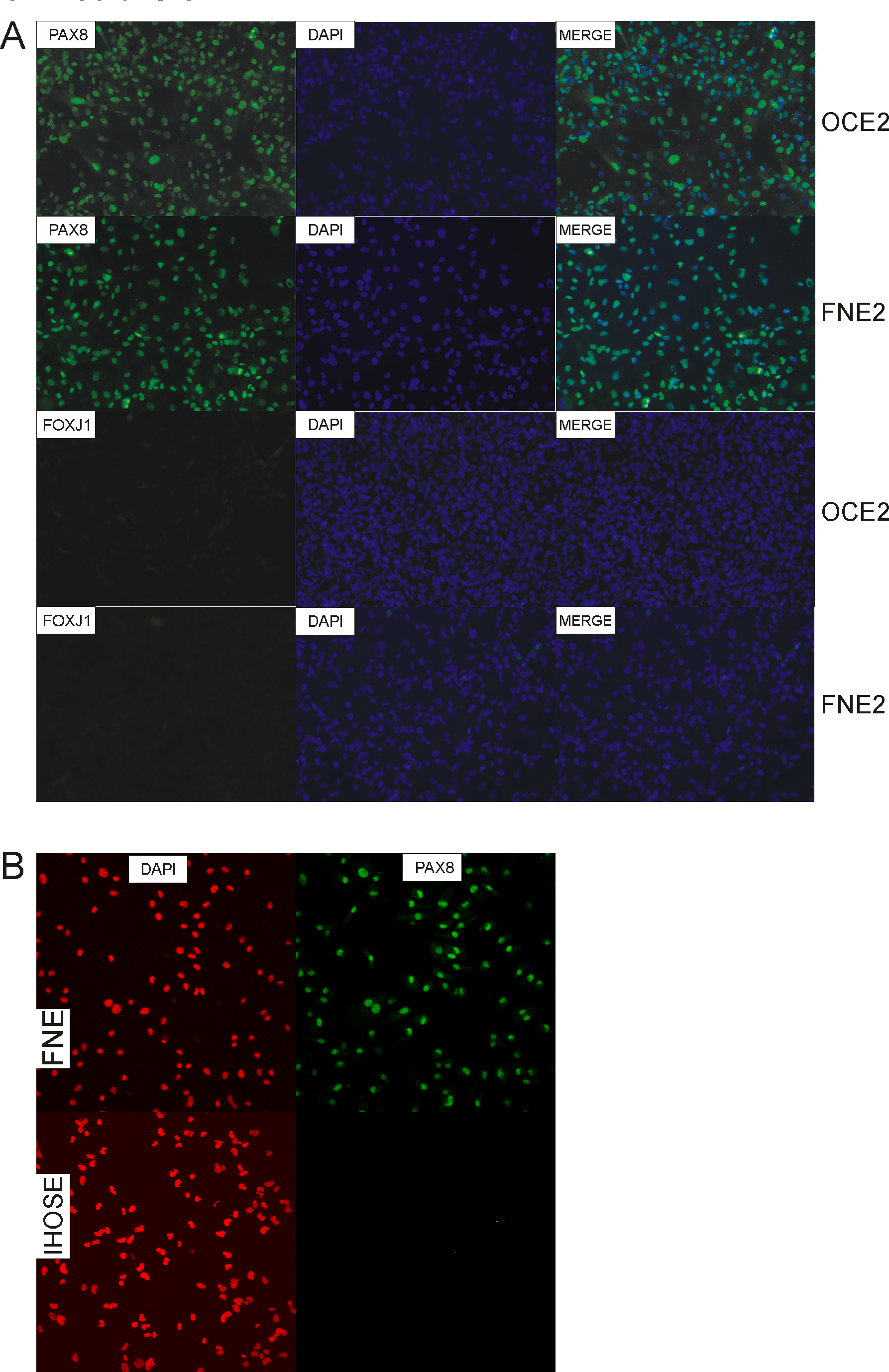

Supplement: Figure S1 — Immunofluorescence staining of cultured OCE and FNE cells for PAX8 and FOXJ1. A-B, Immunofluorescence staining shows that OCE and FNE cells are PAX8+/FOXJ1¯ while IHOSE cells (immortalized using HPV E6/E7 [Tsao et al. 1995, Exp Cell Res 218: 499-507]) were PAX8¯. FNE1 and FNE2 indicate that these cells were derived from patients 1 and 2, respectively. The positive control for FOXJ1 (ciliated pig kidney cells [i.e., LLC-PK1]) all showed positive nuclear staining (data not shown). Experimental conditions for immunofluorescence are detailed in the ‘Antibodies and experimental conditions' table in the Supplementary Methods in File S1. (TIF) [file pone.0080314.s001.tif]

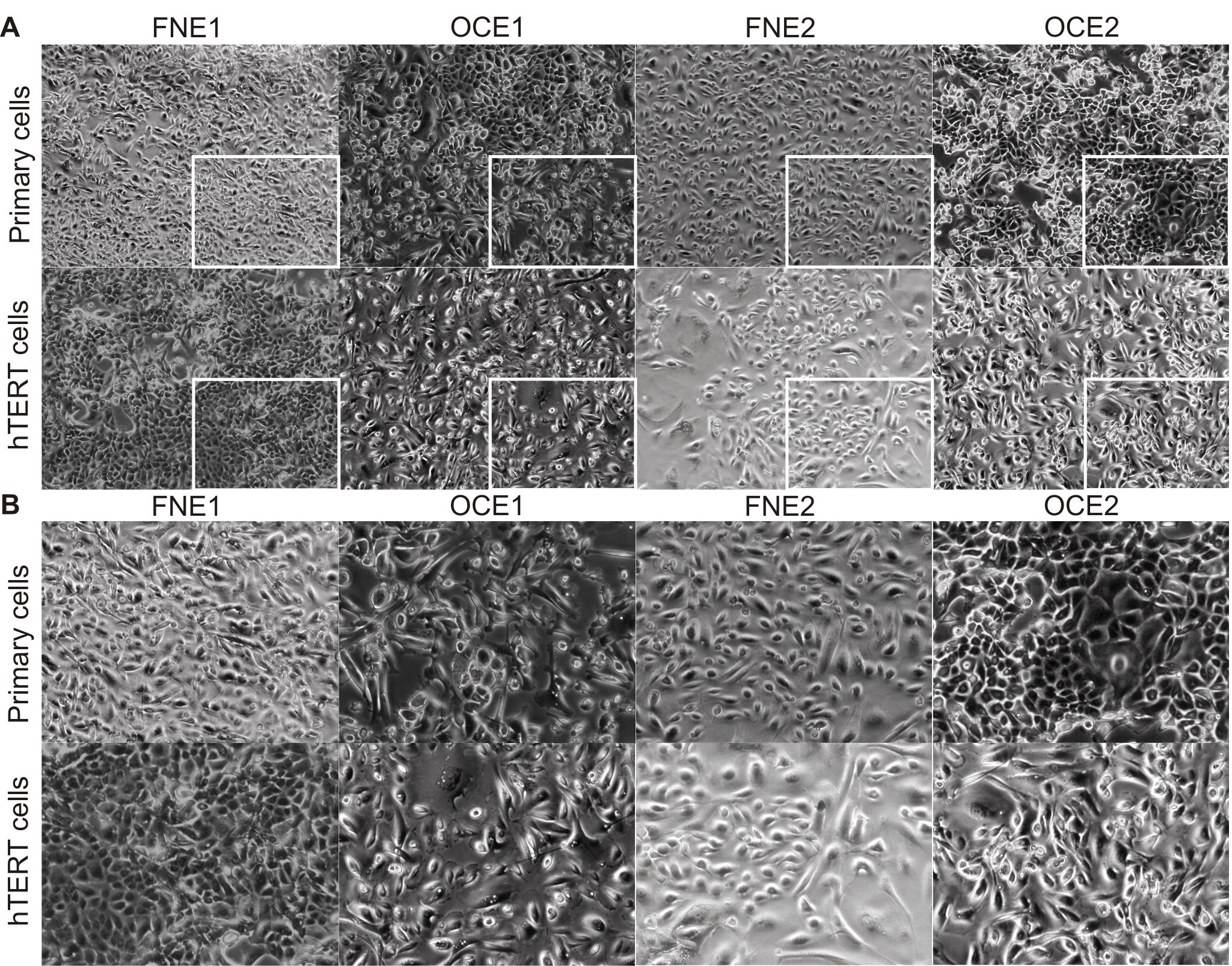

Supplement: Figure S2 — Morphology of primary and hTERT-immortalized FNE and OCE cells in WIT-fo medium. A, Photographs at 10× magnification. B, Cropped and enlarged photographs (white frames in Fig. S2A). (TIF) [file pone.0080314.s002.tif]
