# Supplementary material for: Gene Expression Signature of Normal Cell-of-Origin Predicts Ovarian Tumor Outcomes
Source: PLoS One. 2013 Nov 26;8(11):e80314. doi: 10.1371/journal.pone.0080314 (PMC3841174; doi:10.1371/journal.pone.0080314)
Supplement: Table S1 — Probesets that were up-regulated (n = 632) in comparisons of immortalized fallopian tube non-ciliated epithelium (FNE) versus immortalized ovarian epithelium (OCE) (FDR adjusted P <0.05). (PDF) [file pone.0080314.s003.pdf]

Coef.ft.n...ov.n	t.ft.n...ov.n	p.value.adj.ft.n...ov.n	Res.ft.n...ov.n	ID	gene.symbols
1.704	12.72	1.00E-06	1	214844_s_at	DOK5
0.868	11.73	1.00E-06	1	227259_at	CD47
0.644	11.12	2.00E-06	1	1552842_at	HS6ST3
2.749	11.38	2.00E-06	1	228546_at	DPP6
1.042	10.81	3.00E-06	1	1554863_s_at	DOK5
0.741	10.56	3.00E-06	1	209627_s_at	OSBPL3
1.377	10.28	5.00E-06	1	232276_at	HS6ST3
1.186	10.03	6.00E-06	1	219440_at	RAI2
0.504	9.47	1.30E-05	1	205829_at	HSD17B1
0.692	9.47	1.30E-05	1	221325_at	KCNK13
0.539	9.58	1.30E-05	1	226992_at	NOSTRIN
3.282	9.37	1.40E-05	1	202718_at	IGFBP2
0.597	9.21	1.70E-05	1	213055_at	CD47
0.891	9.08	2.10E-05	1	235085_at	PRAGMIN
0.507	8.95	2.30E-05	1	229952_at	NA
1.178	8.84	2.80E-05	1	229714_at	HS6ST3
0.746	8.77	3.00E-05	1	205593_s_at	PDE9A
0.738	8.73	3.10E-05	1	200644_at	MARCKSL1
1.099	8.67	3.30E-05	1	226016_at	CD47
1.212	8.61	3.70E-05	1	203028_s_at	CYBA
1.343	8.57	3.80E-05	1	233550_s_at	SLC4A11
1.131	8.47	4.30E-05	1	203300_x_at	AP1S2
0.387	8.41	4.60E-05	1	213715_s_at	KANK3
1.005	8.36	4.70E-05	1	206277_at	P2RY2
0.591	8.38	4.70E-05	1	220987_s_at	NA
0.422	8.26	5.30E-05	1	230261_at	ST8SIA4
1.713	8.24	5.40E-05	1	223748_at	SLC4A11
1.413	8.11	6.30E-05	1	207789_s_at	DPP6
2.27	8.11	6.30E-05	1	223385_at	CYP2S1
1.605	8.14	6.30E-05	1	232060_at	ROR1
1.269	8.05	6.90E-05	1	232275_s_at	HS6ST3
1.74	7.95	8.10E-05	1	215789_s_at	AJAP1
1.142	7.89	9.00E-05	1	230264_s_at	AP1S2
0.997	7.86	9.20E-05	1	1558280_s_at	ARHGAP29
0.519	7.78	0.000105	1	205858_at	NGFR
0.516	7.73	0.000109	1	203299_s_at	AP1S2
1.796	7.74	0.000109	1	206460_at	AJAP1
0.637	7.75	0.000109	1	215149_at	NA
0.843	7.69	0.000118	1	1554079_at	GALNTL4
0.627	7.68	0.000118	1	230005_at	SVIP
0.76	7.66	0.000121	1	225792_at	HOOK1
0.533	7.52	0.000151	1	212823_s_at	PLEKHG3
0.72	7.49	0.000159	1	205389_s_at	ANK1
0.887	7.46	0.000166	1	208353_x_at	ANK1
1.097	7.42	0.000176	1	202430_s_at	PLSCR1
2.088	7.39	0.000184	1	228885_at	MAMDC2
0.641	7.36	0.000191	1	230285_at	SVIP
0.502	7.34	0.000196	1	224624_at	LRRC8A
0.604	7.33	2.00E-04	1	205191_at	RP2

Coef.ft.n...ov.n	t.ft.n...ov.n	p.value.adj.ft.n...ov.n	Res.ft.n...ov.n	ID	gene.symbols
0.687	7.23	0.000232	1	216481_at	GRIP2
0.429	7.11	0.00029	1	219157_at	KLHL2
0.815	7.09	0.000301	1	226558_at	NA
0.688	7.05	0.000318	1	228370_at	SNRPN
0.375	7.04	0.000321	1	212750_at	PPP1R16B
0.548	6.99	0.000351	1	220115_s_at	CDH10
0.409	6.93	0.000395	1	228635_at	PCDH10
0.735	6.87	0.000442	1	212651_at	RHOBTB1
0.565	6.86	0.000442	1	222833_at	LPCAT2
0.266	6.86	0.000442	1	227911_at	ARHGAP28
0.602	6.82	0.000472	1	209529_at	PPAP2C
0.237	6.78	0.000498	1	229334_at	RUFY3
0.422	6.77	0.000506	1	219695_at	SMPD3
0.367	6.76	0.000506	1	45749_at	FAM65A
0.516	6.75	0.000511	1	219230_at	TMEM100
0.538	6.69	0.000561	1	204301_at	KBTBD11
0.425	6.69	0.000561	1	222725_s_at	PALMD
0.49	6.66	0.000581	1	212686_at	PPM1H
1.704	6.63	0.000608	1	1563038_at	NA
1.08	6.62	0.000608	1	202446_s_at	PLSCR1
0.966	6.62	0.000608	1	208190_s_at	LSR
0.367	6.62	0.000608	1	213745_at	ATRNL1
0.789	6.59	0.000639	1	236264_at	LPHN3
0.594	6.58	0.000643	1	230006_s_at	SVIP
0.686	6.57	0.000647	1	205390_s_at	ANK1
0.373	6.56	0.000647	1	219024_at	PLEKHA1
0.659	6.54	0.000676	1	225835_at	SLC12A2
0.862	6.52	0.000684	1	202193_at	LIMK2
0.714	6.51	0.000691	1	202133_at	WWTR1
1.128	6.51	0.000691	1	242794_at	MAML3
1.609	6.49	0.000715	1	205805_s_at	ROR1
0.359	6.48	0.000725	1	204302_s_at	KIAA0427
0.213	6.44	0.000762	1	1560676_at	SIAH3
0.798	6.37	0.000872	1	213059_at	CREB3L1
0.468	6.36	0.000876	1	219414_at	CLSTN2
0.306	6.36	0.000876	1	221088_s_at	PPP1R9A
0.893	6.36	0.000876	1	226829_at	AFAP1L2
0.765	6.35	0.000877	1	1553243_at	ITIH5
1.293	6.34	0.000886	1	204567_s_at	ABCG1
0.339	6.34	0.000886	1	207087_x_at	ANK1
0.263	6.33	9.00E-04	1	230836_at	ST8SIA4
0.632	6.32	0.000913	1	209453_at	SLC9A1
0.622	6.31	0.000916	1	229978_at	SHISA9
0.77	6.26	0.001002	1	218736_s_at	PALMD
1.306	6.26	0.001004	1	230831_at	FRMD5
3.407	6.25	0.00102	1	206859_s_at	PAEP
0.81	6.23	0.00104	1	228415_at	AP1S2
0.479	6.22	0.001059	1	204687_at	PARM1
0.703	6.21	0.001084	1	230413_s_at	NA

Coef.ft.n...ov.n	t.ft.n...ov.n	p.value.adj.ft.n...ov.n	Res.ft.n...ov.n	ID	gene.symbols
0.65	6.2	0.001086	1	225660_at	SEMA6A
0.238	6.2	0.001088	1	1553132_a_at	TC2N
0.445	6.18	0.001118	1	218693_at	TSPAN15
0.354	6.17	0.001144	1	221946_at	C9orf116
0.45	6.16	0.001162	1	212255_s_at	ATP2C1
1.803	6.15	0.001183	1	225619_at	SLAIN1
1.025	6.14	0.001183	1	227949_at	PHACTR3
0.417	6.1	0.001292	1	233487_s_at	LRRC8A
0.394	6.09	0.001311	1	232081_at	NA
0.48	6.04	0.001392	1	209197_at	SYT11
0.663	6.04	0.001392	1	214434_at	HSPA12A
0.643	6.04	0.001392	1	221802_s_at	KIAA1598
0.357	6.04	0.001392	1	223823_at	KCNMB2
0.232	6.04	0.001392	1	229623_at	TMEM150C
0.952	6.03	0.001392	1	241456_at	FAM78B
1.626	6.02	0.001401	1	210517_s_at	AKAP12
1.039	6.01	0.001438	1	204540_at	EEF1A2
0.529	5.97	0.001541	1	228201_at	ARL13B
0.217	5.97	0.001541	1	235470_at	NA
0.763	5.95	0.001592	1	225809_at	PARM1
0.998	5.93	0.00165	1	219416_at	SCARA3
0.468	5.93	0.00165	1	223204_at	FAM198B
0.576	5.93	0.001653	1	211113_s_at	ABCG1
0.554	5.92	0.001653	1	221527_s_at	PARD3
0.45	5.89	0.001771	1	213498_at	CREB3L1
1.161	5.88	0.001807	1	225167_at	FRMD4A
0.363	5.87	0.001819	1	226587_at	SNRPN
0.782	5.86	0.001837	1	219064_at	ITIH5
0.632	5.85	0.001898	1	228335_at	CLDN11
1.291	5.82	0.002023	1	206987_x_at	FGF18
0.851	5.81	0.002041	1	202803_s_at	ITGB2
0.625	5.8	0.002111	1	232481_s_at	SLITRK6
0.476	5.77	0.002238	1	200788_s_at	PEA15
0.359	5.76	0.002255	1	227289_at	PCDH17
0.435	5.73	0.002374	1	213172_at	TTC9
0.404	5.7	0.002502	1	218029_at	FAM65A
0.319	5.69	0.002544	1	228863_at	PCDH17
0.353	5.69	0.002544	1	239598_s_at	LPCAT2
0.922	5.66	0.002625	1	212830_at	MEGF9
1.5	5.65	0.002631	1	206385_s_at	ANK3
0.561	5.65	0.002631	1	225147_at	CYTH3
1.438	5.65	0.002635	1	209031_at	CADM1
0.214	5.6	0.0029	1	238747_at	NA
0.282	5.58	0.003081	1	212763_at	CAMSAP1L1
1.444	5.57	0.003126	1	222925_at	DCDC2
0.886	5.55	0.003207	1	227889_at	LPCAT2
0.387	5.53	0.00336	1	204378_at	BCAS1
0.528	5.52	0.003387	1	232176_at	SLITRK6
0.266	5.52	0.003395	1	236128_at	ZNF91

Coef.ft.n...ov.n	t.ft.n...ov.n	p.value.adj.ft.n...ov.n	Res.ft.n...ov.n	ID	gene.symbols
0.322	5.51	0.003395	1	242943_at	ST8SIA4
0.322	5.51	0.003398	1	206497_at	C7orf44
0.475	5.5	0.003506	1	226247_at	PLEKHA1
0.616	5.49	0.003561	1	228494_at	PPP1R9A
1.057	5.49	0.003566	1	225163_at	FRMD4A
0.274	5.48	0.003593	1	241470_x_at	NA
0.415	5.47	0.003623	1	205794_s_at	NOVA1
0.543	5.47	0.003623	1	210017_at	MALT1
0.784	5.47	0.003623	1	213857_s_at	CD47
0.968	5.47	0.003623	1	227318_at	NA
0.163	5.46	0.003734	1	242529_x_at	NA
0.3	5.45	0.003753	1	219660_s_at	ATP8A2
1.132	5.44	0.003846	1	203910_at	ARHGAP29
0.432	5.44	0.003847	1	209155_s_at	NT5C2
1.21	5.43	0.003855	1	211029_x_at	FGF18
0.28	5.43	0.003883	1	207542_s_at	NA
0.262	5.4	0.004088	1	229041_s_at	NA
0.583	5.39	0.004141	1	218311_at	MAP4K3
0.33	5.38	0.00428	1	204477_at	RABIF
1.433	5.36	0.00439	1	220043_s_at	MFI2
1.3	5.35	0.004563	1	204519_s_at	PLLP
0.369	5.34	0.004582	1	213802_at	NA
0.305	5.33	0.004627	1	218318_s_at	NLK
0.395	5.31	0.004853	1	208352_x_at	ANK1
0.439	5.31	0.00487	1	221792_at	NA
0.54	5.31	0.004882	1	204303_s_at	KIAA0427
0.724	5.29	0.005068	1	211075_s_at	CD47
0.537	5.29	0.005073	1	218611_at	IER5
0.335	5.29	0.005073	1	238755_at	NA
0.742	5.28	0.005158	1	35666_at	SEMA3F
0.185	5.28	0.005162	1	207175_at	ADIPOQ
0.334	5.27	0.005196	1	202132_at	WWTR1
0.351	5.24	0.005461	1	210108_at	CACNA1D
0.858	5.22	0.00555	1	206832_s_at	SEMA3F
0.379	5.22	0.00555	1	212765_at	CAMSAP1L1
1.022	5.23	0.00555	1	219778_at	ZFPM2
0.653	5.22	0.00555	1	225525_at	KIAA1671
0.444	5.22	0.00555	1	238044_at	NA
1.93	5.21	0.00559	1	213841_at	NA
0.256	5.21	0.00559	1	228709_at	TPR
0.528	5.21	0.00559	1	229580_at	NA
1.498	5.21	0.00559	1	231382_at	FGF18
1.385	5.2	0.005641	1	205969_at	AADAC
0.98	5.19	0.005722	1	240633_at	DOK7
0.659	5.19	0.005731	1	230913_at	NA
0.541	5.18	0.005812	1	221861_at	NA
0.362	5.18	0.005873	1	226591_at	SNRPN
0.767	5.17	0.005994	1	201522_x_at	NA
0.33	5.16	0.00612	1	213309_at	PLCL2

Coef.ft.n...ov.n	t.ft.n...ov.n	p.value.adj.ft.n...ov.n	Res.ft.n...ov.n	ID	gene.symbols
0.889	5.15	0.006147	1	211485_s_at	FGF18
0.605	5.15	0.006167	1	205302_at	IGFBP1
0.918	5.15	0.006167	1	209568_s_at	RGL1
0.753	5.15	0.006167	1	213039_at	ARHGEF18
0.484	5.14	0.006244	1	223535_at	NUDT12
0.531	5.14	0.00628	1	204925_at	CTNS
0.346	5.13	0.00636	1	205391_x_at	ANK1
1.999	5.13	0.00636	1	235911_at	LOC440995
0.775	5.12	0.006368	1	212530_at	NEK7
0.809	5.12	0.006385	1	229927_at	LEMD1
0.596	5.11	0.006518	1	215790_at	AJAP1
0.661	5.11	0.006543	1	235150_at	NA
1.033	5.1	0.006621	1	209032_s_at	CADM1
0.945	5.09	0.006747	1	222802_at	EDN1
0.62	5.07	0.006989	1	225020_at	DAB2IP
0.592	5.06	0.00701	1	1569470_a_at	FRMD5
0.44	5.06	0.00701	1	222819_at	CTPS2
0.284	5.06	0.00705	1	231732_at	SMPD3
0.383	5.06	0.007064	1	211137_s_at	ATP2C1
0.435	5.05	0.007147	1	212321_at	SGPL1
0.758	5.05	0.007172	1	201236_s_at	BTG2
0.71	5.04	0.0073	1	217475_s_at	LIMK2
0.447	5.03	0.007311	1	222589_at	NLK
0.537	5.03	0.007341	1	225868_at	TRIM47
0.776	5.03	0.007364	1	206042_x_at	NA
0.342	5.02	0.007364	1	230174_at	LYPLAL1
0.977	5.02	0.007364	1	239579_at	EPHX4
0.659	5	0.007754	1	204200_s_at	PDGFB
0.536	5	0.007804	1	222581_at	XPR1
0.894	4.99	0.007833	1	238206_at	RXFP1
0.401	4.99	0.007896	1	204364_s_at	REEP1
0.308	4.97	0.008149	1	235684_s_at	SESN3
0.476	4.97	0.008153	1	58780_s_at	FLJ10357
0.657	4.96	0.008184	1	227410_at	FAM43A
0.385	4.96	0.008213	1	232125_at	NA
0.591	4.96	0.008231	1	209730_at	SEMA3F
0.202	4.96	0.008231	1	209795_at	CD69
0.423	4.95	0.00837	1	212831_at	MEGF9
0.37	4.94	0.0086	1	202804_at	ABCC1
0.468	4.94	0.008645	1	225447_at	GPD2
0.543	4.92	0.008883	1	235775_at	TMTC2
0.463	4.92	0.008912	1	225627_s_at	CACHD1
2.043	4.91	0.008921	1	209074_s_at	FAM107A
0.44	4.9	0.009107	1	1552754_a_at	CADM2
0.293	4.89	0.009344	1	226688_at	C3orf23
0.385	4.89	0.009355	1	203221_at	TLE1
0.614	4.89	0.009359	1	210740_s_at	ITPK1
1.443	4.87	0.009568	1	227530_at	AKAP12
0.307	4.85	0.009918	1	221280_s_at	PARD3

Coef.ft.n...ov.n	t.ft.n...ov.n	p.value.adj.ft.n...ov.n	Res.ft.n...ov.n	ID	gene.symbols
1.105	4.82	0.010536	1	209030_s_at	CADM1
0.9	4.82	0.010597	1	231804_at	RXFP1
0.161	4.82	0.010639	1	233976_at	NA
0.483	4.82	0.010666	1	214053_at	ERBB4
1.547	4.81	0.010774	1	227529_s_at	AKAP12
0.283	4.8	0.010874	1	242245_at	NA
0.388	4.8	0.011026	1	202187_s_at	PPP2R5A
0.469	4.8	0.011026	1	229380_at	NA
0.671	4.79	0.011042	1	204679_at	KCNK1
0.823	4.79	0.011042	1	221974_at	IPW
0.382	4.77	0.011471	1	221458_at	HTR1F
0.274	4.77	0.011606	1	225313_at	C20orf177
0.516	4.76	0.011785	1	212800_at	STX6
0.187	4.75	0.012002	1	1556328_at	NA
0.597	4.75	0.012002	1	214284_s_at	FGF18
0.195	4.75	0.012002	1	230534_at	ZNF678
0.296	4.74	0.012163	1	201910_at	FARP1
0.286	4.74	0.01221	1	222696_at	AXIN2
0.352	4.73	0.012239	1	207981_s_at	ESRRG
0.443	4.73	0.012239	1	221606_s_at	HMGN5
0.266	4.73	0.012358	1	219384_s_at	ADAT1
0.252	4.73	0.012363	1	227984_at	LOC650392
0.203	4.71	0.012741	1	227929_at	NA
0.508	4.7	0.012878	1	36566_at	CTNS
1.011	4.7	0.01299	1	209792_s_at	KLK10
0.32	4.69	0.013216	1	218981_at	ACN9
0.498	4.69	0.013216	1	49111_at	NA
2.79	4.68	0.013391	1	204475_at	MMP1
0.376	4.68	0.013469	1	209198_s_at	SYT11
0.393	4.67	0.013532	1	233528_s_at	NA
0.345	4.67	0.013603	1	205122_at	TMEFF1
0.69	4.67	0.013603	1	209442_x_at	ANK3
0.338	4.65	0.014033	1	218676_s_at	PCTP
2.856	4.65	0.014072	1	206658_at	UPK3B
0.841	4.64	0.014132	1	219045_at	RHOF
0.735	4.64	0.014145	1	211555_s_at	GUCY1B3
0.246	4.64	0.014145	1	222631_at	PI4K2B
0.231	4.63	0.014383	1	216869_at	PDE1C
0.224	4.63	0.014491	1	204672_s_at	ANKRD6
0.582	4.63	0.014491	1	212799_at	STX6
1.286	4.62	0.014589	1	205680_at	MMP10
0.598	4.62	0.014607	1	213174_at	TTC9
0.574	4.62	0.014738	1	1555310_a_at	PAK6
0.494	4.61	0.014799	1	242186_x_at	LPHN3
0.428	4.61	0.014829	1	227121_at	NA
0.333	4.6	0.015132	1	223063_at	C1orf198
0.892	4.6	0.015189	1	218417_s_at	SLC48A1
2.313	4.59	0.015259	1	204468_s_at	TIE1
1.84	4.58	0.015503	1	205479_s_at	PLAU

Coef.ft.n...ov.n	t.ft.n...ov.n	p.value.adj.ft.n...ov.n	Res.ft.n...ov.n	ID	gene.symbols
0.675	4.58	0.015592	1	205258_at	INHBB
0.337	4.58	0.015686	1	203144_s_at	KIAA0040
1.846	4.57	0.015757	1	211668_s_at	PLAU
1.319	4.56	0.015967	1	223842_s_at	SCARA3
0.318	4.55	0.016269	1	217196_s_at	CAMSAP1L1
0.52	4.55	0.016273	1	222240_s_at	ISYNA1
0.582	4.55	0.016347	1	219619_at	DIRAS2
0.298	4.55	0.016347	1	230183_at	EXT1
0.509	4.55	0.016347	1	238722_x_at	NAPEPLD
0.21	4.55	0.016347	1	242919_at	NA
0.497	4.54	0.016488	1	235333_at	B4GALT6
0.332	4.54	0.016497	1	202805_s_at	ABCC1
0.892	4.53	0.016564	1	219274_at	TSPAN12
0.519	4.53	0.016568	1	208476_s_at	FRMD4A
0.636	4.53	0.016568	1	210582_s_at	LIMK2
0.592	4.53	0.016568	1	217853_at	TNS3
1.068	4.53	0.016599	1	226884_at	LRRN1
0.626	4.52	0.016919	1	212664_at	TUBB4
0.342	4.51	0.01718	1	206232_s_at	B4GALT6
1.111	4.51	0.01718	1	231067_s_at	NA
0.399	4.51	0.017305	1	218826_at	SLC35F2
0.155	4.49	0.017788	1	231026_at	EFHC1
0.444	4.49	0.018013	1	201235_s_at	BTG2
0.691	4.48	0.018043	1	211663_x_at	PTGDS
0.303	4.48	0.018065	1	229579_s_at	DISP2
0.137	4.48	0.018165	1	237667_at	NA
0.294	4.48	0.018179	1	220992_s_at	C1orf25
0.451	4.48	0.018179	1	43511_s_at	NA
0.565	4.47	0.018181	1	226259_at	EXOC6
0.187	4.47	0.018282	1	1553726_s_at	C6orf170
0.588	4.47	0.018368	1	236344_at	PDE1C
1.395	4.46	0.018692	1	204698_at	ISG20
1.555	4.46	0.018692	1	206697_s_at	HP
0.357	4.46	0.018692	1	219696_at	DENND1B
0.355	4.46	0.018692	1	227725_at	ST6GALNAC1
0.225	4.46	0.018692	1	227802_at	RUFY3
0.618	4.45	0.01891	1	209935_at	ATP2C1
1.486	4.45	0.019017	1	206884_s_at	SCEL
0.133	4.44	0.019079	1	1556436_at	C8orf50
0.436	4.44	0.019079	1	219659_at	ATP8A2
0.157	4.44	0.019079	1	219976_at	HOOK1
0.146	4.44	0.019079	1	220220_at	LRRC37A4
0.332	4.44	0.019079	1	227180_at	ELOVL7
0.526	4.44	0.019079	1	229634_at	TMEM139
0.236	4.44	0.019079	1	230176_at	NA
0.62	4.44	0.019079	1	239155_at	NA
0.776	4.42	0.019621	1	225168_at	FRMD4A
0.187	4.42	0.019638	1	202694_at	STK17A
0.675	4.42	0.019638	1	203408_s_at	SATB1

Coef.ft.n...ov.n	t.ft.n...ov.n	p.value.adj.ft.n...ov.n	Res.ft.n...ov.n	ID	gene.symbols
0.574	4.42	0.019647	1	218694_at	ARMCX1
1.149	4.42	0.01969	1	214797_s_at	CDK18
0.209	4.41	0.0198	1	229956_at	NR2C1
0.455	4.41	0.019972	1	225347_at	ARL8A
0.241	4.4	0.020042	1	219248_at	THUMPD2
0.285	4.4	0.020101	1	227040_at	NHLRC3
1.155	4.4	0.020173	1	207547_s_at	FAM107A
0.593	4.4	0.02027	1	213518_at	PRKCI
0.395	4.39	0.020475	1	226636_at	PLD1
0.632	4.38	0.02079	1	223342_at	RRM2B
0.282	4.38	0.020821	1	204573_at	CROT
0.454	4.38	0.020821	1	213993_at	SPON1
0.444	4.38	0.020821	1	40284_at	FOXA2
0.364	4.38	0.02084	1	212510_at	GPD1L
0.146	4.38	0.02084	1	220884_at	NA
0.194	4.37	0.02089	1	219676_at	ZSCAN16
0.611	4.37	0.020957	1	226278_at	SVIP
0.191	4.37	0.02098	1	34449_at	CASP2
0.235	4.37	0.02103	1	226908_at	LRIG3
0.181	4.36	0.021134	1	221400_at	MYO3A
0.799	4.36	0.02115	1	203817_at	GUCY1B3
0.45	4.36	0.021307	1	207358_x_at	MACF1
0.82	4.35	0.021386	1	1552715_a_at	RXFP1
0.639	4.35	0.021508	1	227230_s_at	KIAA1211
0.258	4.34	0.021781	1	214441_at	STX6
0.298	4.34	0.021814	1	218928_s_at	SLC37A1
0.523	4.33	0.022181	1	211833_s_at	BAX
0.229	4.33	0.022204	1	238532_at	DPF3
0.165	4.33	0.022204	1	243501_at	NA
0.315	4.33	0.022248	1	204249_s_at	LMO2
0.358	4.33	0.022248	1	226066_at	MITF
0.176	4.33	0.022396	1	1562699_at	NA
0.206	4.32	0.022491	1	1554034_a_at	FRMD4A
0.248	4.32	0.022491	1	223511_at	C1orf124
0.171	4.32	0.022491	1	240433_x_at	NA
0.667	4.32	0.022521	1	218313_s_at	GALNT7
0.205	4.32	0.022521	1	223611_s_at	LNX1
0.111	4.32	0.022521	1	232022_at	NA
1.221	4.32	0.022521	1	232056_at	SCEL
0.46	4.32	0.022549	1	213447_at	NA
0.714	4.31	0.022597	1	211990_at	HLA-DPA1
0.487	4.31	0.022601	1	1557531_a_at	C10orf55
0.596	4.31	0.022671	1	226041_at	NAPEPLD
0.367	4.31	0.022691	1	242906_at	NA
0.294	4.3	0.022806	1	226615_at	XPR1
0.356	4.3	0.022808	1	206523_at	CYTH3
0.673	4.3	0.022821	1	225602_at	GLIPR2
0.155	4.3	0.022821	1	233658_at	NA
0.852	4.3	0.022913	1	219143_s_at	RPP25

Coef.ft.n...ov.n	t.ft.n...ov.n	p.value.adj.ft.n...ov.n	Res.ft.n...ov.n	ID	gene.symbols
0.168	4.3	0.022913	1	243542_at	PREPL
0.4	4.29	0.023323	1	1555349_a_at	ITGB2
0.516	4.28	0.023782	1	204678_s_at	KCNK1
0.17	4.28	0.023782	1	214834_at	PAR5
0.513	4.28	0.023782	1	235308_at	ZBTB20
0.228	4.28	0.023782	1	239818_x_at	TRIB1
0.626	4.28	0.023782	1	242406_at	NA
0.398	4.27	0.023939	1	204781_s_at	FAS
0.667	4.27	0.024164	1	209803_s_at	PHLDA2
0.214	4.27	0.024203	1	220888_s_at	CASS4
0.439	4.26	0.024234	1	218995_s_at	EDN1
0.407	4.26	0.024234	1	242635_s_at	NAPEPLD
0.159	4.26	0.024292	1	1554985_at	ZNF396
0.278	4.26	0.024397	1	204496_at	STRN3
0.189	4.26	0.024435	1	220363_s_at	ELMO2
0.289	4.26	0.024435	1	59437_at	C9orf116
0.714	4.25	0.02468	1	201811_x_at	SH3BP5
0.282	4.24	0.025438	1	235976_at	SLITRK6
0.139	4.23	0.025889	1	1570213_at	NA
0.233	4.22	0.025943	1	206299_at	FAM155B
0.303	4.22	0.025943	1	213074_at	PHIP
0.224	4.22	0.025943	1	222520_s_at	IFT57
0.424	4.22	0.025943	1	243764_at	VSIG1
0.557	4.21	0.026284	1	206595_at	CST6
0.208	4.21	0.026297	1	238799_at	ATP5S
0.455	4.21	0.026475	1	235044_at	CYYR1
0.824	4.21	0.026475	1	235165_at	PARD6B
0.782	4.21	0.026489	1	236088_at	NTNG1
0.274	4.21	0.026508	1	221796_at	NTRK2
0.248	4.2	0.026594	1	1556209_at	CLEC2B
0.439	4.2	0.026594	1	208634_s_at	MACF1
0.223	4.2	0.026781	1	227715_at	NA
0.204	4.2	0.026879	1	206723_s_at	LPAR2
0.299	4.19	0.027324	1	228437_at	CNIH4
0.494	4.19	0.027456	1	227196_at	RHPN2
0.714	4.18	0.027501	1	201889_at	FAM3C
0.303	4.18	0.027501	1	226000_at	CTTNBP2NL
0.147	4.18	0.02757	1	1556654_at	NA
0.535	4.18	0.02757	1	202241_at	TRIB1
0.407	4.18	0.027701	1	206233_at	B4GALT6
0.276	4.18	0.027701	1	206925_at	ST8SIA4
0.42	4.17	0.027843	1	208478_s_at	BAX
0.663	4.17	0.027941	1	222587_s_at	GALNT7
0.445	4.17	0.028109	1	242899_at	NA
0.109	4.16	0.028477	1	243028_x_at	NA
0.168	4.16	0.028565	1	228867_at	TATDN3
0.315	4.15	0.029015	1	216218_s_at	PLCL2
0.312	4.14	0.029531	1	211122_s_at	CXCL11
1.594	4.14	0.029639	1	204446_s_at	ALOX5

Coef.ft.n...ov.n	t.ft.n...ov.n	p.value.adj.ft.n...ov.n	Res.ft.n...ov.n	ID	gene.symbols
0.47	4.14	0.029639	1	221523_s_at	RRAGD
0.176	4.14	0.029639	1	238451_at	MPP7
0.223	4.13	0.029817	1	238493_at	ZNF506
0.257	4.13	0.029825	1	1569872_a_at	LOC650392
1.137	4.13	0.029825	1	223503_at	TMEM163
0.145	4.13	0.030042	1	227297_at	ITGA9
0.203	4.12	0.030186	1	213537_at	HLA-DPA1
0.348	4.12	0.030463	1	210018_x_at	MALT1
0.407	4.12	0.030463	1	230084_at	SLC30A2
1.13	4.11	0.030841	1	210815_s_at	CALCRL
0.561	4.11	0.030841	1	227231_at	KIAA1211
0.253	4.11	0.031001	1	55692_at	ELMO2
0.195	4.11	0.031044	1	1556619_at	SHISA9
0.234	4.11	0.031074	1	1560318_at	ARHGAP29
0.463	4.1	0.031083	1	241014_at	LOC339400
0.176	4.1	0.031431	1	214586_at	GPR37
0.262	4.09	0.031563	1	235051_at	CCDC50
0.391	4.1	0.031563	1	235230_at	NA
1.183	4.09	0.0317	1	206825_at	OXTR
1.035	4.08	0.032051	1	211748_x_at	PTGDS
2.203	4.09	0.032051	1	213317_at	CLIC5
0.469	4.08	0.032534	1	228101_at	APBA1
0.305	4.07	0.032692	1	225810_at	MTMR10
0.222	4.07	0.033014	1	231395_at	ATP8A2
0.438	4.07	0.033171	1	223449_at	SEMA6A
0.639	4.06	0.033435	1	234996_at	CALCRL
0.629	4.06	0.033591	1	213523_at	CCNE1
0.869	4.05	0.034067	1	1554921_a_at	SCEL
0.264	4.05	0.034313	1	222871_at	KLHDC8A
1.021	4.05	0.034316	1	225123_at	NA
0.216	4.05	0.034316	1	235970_at	LCORL
0.211	4.05	0.034349	1	204129_at	BCL9
0.394	4.05	0.034349	1	226674_at	SHISA4
0.229	4.05	0.034349	1	230237_at	ADCYAP1
0.316	4.04	0.034632	1	211991_s_at	HLA-DPA1
0.224	4.04	0.034632	1	229313_at	ANO5
1.177	4.04	0.034632	1	33304_at	ISG20
0.251	4.03	0.035	1	226869_at	MEGF6
0.204	4.03	0.035	1	244364_at	MYO3A
0.331	4.03	0.03506	1	202321_at	GGPS1
1.018	4.03	0.035242	1	212338_at	MYO1D
0.708	4.03	0.035279	1	209866_s_at	LPHN3
0.196	4.03	0.035331	1	207103_at	KCND2
0.503	4.02	0.035356	1	209993_at	ABCB1
0.604	4.02	0.035431	1	211518_s_at	BMP4
0.686	4.02	0.035952	1	212560_at	SORL1
0.256	4.01	0.036388	1	228600_x_at	C7orf46
0.203	4.01	0.036504	1	221614_s_at	RPH3AL
0.183	4	0.036607	1	1564630_at	EDN1

Coef.ft.n...ov.n	t.ft.n...ov.n	p.value.adj.ft.n...ov.n	Res.ft.n...ov.n	ID	gene.symbols
0.453	4	0.036735	1	212111_at	STX12
0.152	4	0.036895	1	1569277_at	NA
0.126	4	0.036895	1	235943_at	FLJ13197
0.211	3.99	0.036941	1	239229_at	NA
0.961	3.99	0.036997	1	205659_at	HDAC9
0.143	3.99	0.037016	1	210272_at	CYP2B7P1
0.221	3.99	0.037198	1	228574_at	TMTC2
0.21	3.99	0.037198	1	229695_at	NA
0.608	3.99	0.037228	1	222812_s_at	RHOF
0.298	3.99	0.037495	1	238323_at	TEAD2
0.209	3.98	0.037694	1	1554333_at	DNAJA4
0.939	3.98	0.037858	1	211480_s_at	SLCO1A2
0.574	3.98	0.037858	1	213158_at	NA
0.536	3.98	0.037858	1	227347_x_at	HES4
0.203	3.98	0.037858	1	243582_at	SH3RF2
0.217	3.98	0.037935	1	221528_s_at	ELMO2
0.101	3.98	0.037943	1	215410_at	NA
0.218	3.97	0.038179	1	1555781_at	PQLC2
0.309	3.97	0.038179	1	203222_s_at	TLE1
0.675	3.97	0.038179	1	227753_at	TMEM139
0.242	3.97	0.038179	1	237169_at	NA
0.457	3.97	0.038291	1	212399_s_at	VGLL4
0.529	3.97	0.038291	1	227533_at	NA
0.128	3.97	0.038335	1	208375_at	IFNA1
0.304	3.96	0.03841	1	230968_at	NA
0.167	3.96	0.03841	1	242548_x_at	ANKRD37
0.181	3.96	0.038714	1	207233_s_at	MITF
0.529	3.96	0.038714	1	225421_at	PM20D2
0.183	3.95	0.038885	1	236527_at	NA
0.183	3.95	0.038885	1	243077_at	FLJ16734
0.132	3.95	0.039006	1	1554547_at	FAM13C
0.253	3.95	0.039006	1	1555907_at	hCG_2014417
0.662	3.95	0.039006	1	209994_s_at	NA
0.266	3.95	0.039006	1	223415_at	RPP25
0.8	3.95	0.039081	1	225604_s_at	GLIPR2
0.459	3.94	0.039224	1	231183_s_at	JAG1
0.163	3.94	0.039468	1	217391_x_at	NA
0.268	3.94	0.039818	1	218301_at	RNPEPL1
0.157	3.93	0.039882	1	205848_at	GAS2
1.293	3.93	0.039959	1	222281_s_at	NA
1.422	3.93	0.039988	1	209443_at	SERPINA5
0.177	3.93	0.039988	1	235170_at	ZNF92
0.196	3.92	0.040358	1	235447_at	TRUB1
0.125	3.92	0.040534	1	1555071_at	TLL1
0.413	3.92	0.040534	1	208309_s_at	MALT1
0.345	3.92	0.040534	1	226408_at	TEAD2
0.298	3.92	0.040549	1	224534_at	KREMEN1
1.143	3.91	0.04114	1	1555997_s_at	IGFBP5
0.129	3.91	0.041142	1	237552_at	NA

Coef.ft.n...ov.n	t.ft.n...ov.n	p.value.adj.ft.n...ov.n	Res.ft.n...ov.n	ID	gene.symbols
0.246	3.91	0.041201	1	205522_at	HOXD4
0.175	3.91	0.041201	1	238336_s_at	DNAJC21
0.137	3.9	0.041388	1	236489_at	NA
0.212	3.9	0.041656	1	209811_at	CASP2
0.291	3.9	0.041656	1	225068_at	KLHL12
0.197	3.9	0.041656	1	227358_at	ZBTB46
0.459	3.9	0.041859	1	200787_s_at	PEA15
1.016	3.9	0.041859	1	209099_x_at	JAG1
0.349	3.9	0.041859	1	228810_at	CCNYL1
0.291	3.89	0.041938	1	1554762_a_at	WWC2
0.37	3.89	0.041991	1	202514_at	DLG1
0.554	3.89	0.042057	1	209626_s_at	OSBPL3
0.299	3.89	0.04215	1	228221_at	SLC44A3
0.207	3.89	0.042165	1	1558501_at	DNM3
0.117	3.89	0.042267	1	207998_s_at	CACNA1D
0.41	3.89	0.042317	1	215719_x_at	FAS
0.323	3.88	0.0424	1	218304_s_at	OSBPL11
0.311	3.88	0.0424	1	227444_at	ARMCX4
0.14	3.88	0.0424	1	240027_at	LIN7A
0.315	3.88	0.042742	1	220999_s_at	CYFIP2
0.814	3.88	0.042742	1	224707_at	C5orf32
0.266	3.88	0.042876	1	219355_at	CXorf57
1.307	3.88	0.042884	1	215704_at	FLG
0.181	3.87	0.042944	1	206626_x_at	SSX1
0.507	3.87	0.043149	1	215723_s_at	PLD1
0.557	3.87	0.04315	1	219058_x_at	TINAGL1
0.982	3.86	0.043565	1	212187_x_at	PTGDS
0.195	3.86	0.043783	1	242312_x_at	NA
0.275	3.86	0.043792	1	238787_at	DENND1B
0.383	3.86	0.043812	1	229523_at	TMEM200C
0.122	3.86	0.043812	1	236263_at	SHH
1.123	3.85	0.044211	1	226302_at	ATP8B1
0.092	3.85	0.044222	1	1565621_at	NA
0.411	3.85	0.044222	1	242313_at	LOC728730
0.259	3.85	0.044323	1	219296_at	ZDHHC13
0.451	3.85	0.044503	1	204780_s_at	FAS
0.343	3.84	0.044822	1	220326_s_at	FLJ10357
0.169	3.84	0.044829	1	203220_s_at	TLE1
0.607	3.84	0.044975	1	1555963_x_at	B3GNT7
0.492	3.84	0.045021	1	200602_at	APP
0.296	3.84	0.045021	1	203327_at	IDE
0.135	3.84	0.045021	1	240137_at	NA
0.303	3.84	0.045021	1	41577_at	PPP1R16B
0.708	3.84	0.045066	1	1554539_a_at	RHOF
0.605	3.84	0.045066	1	213135_at	TIAM1
1.364	3.83	0.045693	1	211959_at	IGFBP5
0.474	3.83	0.045693	1	212774_at	ZNF238
0.822	3.83	0.045693	1	213994_s_at	SPON1
0.88	3.83	0.0457	1	201340_s_at	ENC1

Coef.ft.n...ov.n	t.ft.n...ov.n	p.value.adj.ft.n...ov.n	Res.ft.n...ov.n	ID	gene.symbols
0.703	3.83	0.045759	1	224901_at	SCD5
1.228	3.82	0.045948	1	219655_at	C7orf10
0.294	3.82	0.045948	1	226338_at	TMEM55A
0.215	3.83	0.045948	1	227777_at	C10orf18
0.412	3.83	0.045948	1	229144_at	RP1-21O18.1
0.143	3.82	0.045948	1	241114_s_at	NA
0.102	3.82	0.046025	1	225630_at	EEPD1
0.8	3.82	0.046089	1	201363_s_at	IVNS1ABP
0.407	3.82	0.046426	1	204343_at	ABCA3
0.411	3.82	0.046426	1	219858_s_at	MFSD6
0.232	3.81	0.046443	1	226537_at	HINT3
0.267	3.81	0.046443	1	227618_at	NA
0.09	3.81	0.046671	1	1566995_at	NA
0.936	3.81	0.046693	1	1552626_a_at	TMEM163
0.441	3.81	0.046771	1	204544_at	HPS5
0.245	3.81	0.046913	1	228790_at	FAM110B
0.49	3.81	0.046963	1	222274_at	ZDHHC8P1
0.209	3.8	0.046997	1	1568603_at	CADPS
0.261	3.8	0.046997	1	221756_at	PIK3IP1
0.484	3.8	0.047128	1	219461_at	PAK6
0.223	3.8	0.047253	1	224176_s_at	AXIN2
0.433	3.8	0.047279	1	212322_at	SGPL1
0.158	3.8	0.047279	1	217566_s_at	TGM4
0.113	3.8	0.047279	1	240828_at	NA
0.304	3.8	0.047389	1	223404_s_at	C1orf25
0.28	3.8	0.04742	1	226380_at	PTPN21
0.202	3.79	0.047613	1	212040_at	TGOLN2
0.122	3.79	0.047781	1	237996_at	NA
0.277	3.79	0.047905	1	203636_at	MID1
0.119	3.79	0.047923	1	1562856_at	NA
0.161	3.79	0.047923	1	243779_at	GALNT13
0.246	3.79	0.048104	1	232130_at	NA
0.109	3.78	0.048259	1	232355_at	SNORD114-3
0.218	3.78	0.048292	1	202744_at	SLC20A2
0.853	3.78	0.048292	1	206331_at	CALCRL
0.243	3.78	0.048332	1	221014_s_at	RAB33B
0.452	3.78	0.04839	1	238617_at	NA
1.209	3.78	0.048431	1	209071_s_at	RGS5
0.331	3.78	0.048657	1	204478_s_at	RABIF
0.302	3.77	0.048887	1	202823_at	TCEB1
0.19	3.77	0.048921	1	205771_s_at	AKAP7
0.178	3.77	0.049024	1	228945_s_at	SLC39A8
1.181	3.77	0.049483	1	217628_at	CLIC5
0.129	3.77	0.049483	1	220813_at	CYSLTR2
