# Supplementary material for: Gene Expression Signature of Normal Cell-of-Origin Predicts Ovarian Tumor Outcomes
Source: PLoS One. 2013 Nov 26;8(11):e80314. doi: 10.1371/journal.pone.0080314 (PMC3841174; doi:10.1371/journal.pone.0080314)
Supplement: Table S2 — Probesets that were up-regulated (n = 525) in immortalized ovarian epithelium (OCE) as compared with immortalized fallopian tube non-ciliated epithelium (FNE) cells (FDR adjusted P <0.05). (PDF) [file pone.0080314.s004.pdf]

Coef.ft.n...ov.n	t.ft.n...ov.n	p.value.adj.ft.n...ov.n	Res.ft.n...ov.n	ID	gene.symbols
-1.449	-10.17	5.00E-06	-1	203438_at	STC2
-1.707	-9.23	1.70E-05	-1	202035_s_at	SFRP1
-0.954	-9.04	2.10E-05	-1	229065_at	SLC35F3
-2.075	-8.51	4.10E-05	-1	202037_s_at	SFRP1
-2.147	-8.37	4.70E-05	-1	202036_s_at	SFRP1
-1.416	-8.33	4.80E-05	-1	203439_s_at	STC2
-1.053	-8.13	6.30E-05	-1	214437_s_at	SHMT2
-0.961	-8	7.50E-05	-1	220486_x_at	TMEM164
-0.958	-7.86	9.20E-05	-1	214096_s_at	SHMT2
-0.963	-7.64	0.000123	-1	223201_s_at	TMEM164
-1.085	-7.62	0.000126	-1	202998_s_at	LOXL2
-0.372	-7.54	0.000146	-1	228028_at	FAM59B
-0.329	-7.4	0.000184	-1	205608_s_at	ANGPT1
-0.927	-7.31	0.000204	-1	223202_s_at	TMEM164
-0.375	-7.27	0.000217	-1	210571_s_at	CMAH
-1.079	-7.23	0.000232	-1	231736_x_at	MGST1
-0.54	-7.16	0.000269	-1	205518_s_at	CMAH
-0.699	-7.12	0.000287	-1	213568_at	OSR2
-1.032	-6.99	0.000351	-1	202765_s_at	FBN1
-1.007	-6.98	0.000351	-1	224918_x_at	MGST1
-0.422	-6.83	0.000463	-1	210999_s_at	GRB10
-1.378	-6.78	0.000498	-1	202887_s_at	DDIT4
-0.349	-6.78	0.000498	-1	209410_s_at	GRB10
-0.951	-6.73	0.000536	-1	214439_x_at	BIN1
-0.958	-6.7	0.000556	-1	210202_s_at	BIN1
-1.014	-6.69	0.000561	-1	213005_s_at	KANK1
-0.457	-6.67	0.000581	-1	213509_x_at	CES2
-0.942	-6.65	0.000584	-1	210201_x_at	BIN1
-0.803	-6.58	0.000643	-1	202997_s_at	LOXL2
-0.516	-6.57	0.000646	-1	235318_at	FBN1
-0.39	-6.57	0.000647	-1	201922_at	NSA2
-1.645	-6.52	0.000684	-1	204284_at	PPP1R3C
-0.579	-6.52	0.000684	-1	46270_at	UBAP1
-0.95	-6.47	0.000743	-1	202931_x_at	BIN1
-1.098	-6.46	0.000746	-1	1565162_s_at	MGST1
-0.809	-6.45	0.000762	-1	200779_at	ATF4
-0.681	-6.43	0.000775	-1	214095_at	SHMT2
-0.361	-6.39	0.000846	-1	232015_at	FAM59B
-0.311	-6.36	0.000876	-1	239315_at	FAM115C
-0.545	-6.34	0.000886	-1	204093_at	CCNH
-0.585	-6.34	0.000886	-1	207180_s_at	HTATIP2
-1.24	-6.31	0.000916	-1	222774_s_at	NETO2
-0.444	-6.27	0.000982	-1	201653_at	CNIH
-0.521	-6.24	0.001032	-1	209739_s_at	PNPLA4
-0.534	-6.23	0.001054	-1	202143_s_at	COPS8

Coef.ft.n...ov.n	t.ft.n...ov.n	p.value.adj.ft.n...ov.n	Res.ft.n...ov.n	ID	gene.symbols
-0.292	-6.2	0.001091	-1	208780_x_at	VAPA
-1.344	-6.17	0.001144	-1	218888_s_at	NETO2
-0.892	-6.08	0.001333	-1	205100_at	GFPT2
-0.556	-6.08	0.001333	-1	221775_x_at	RPL22
-0.356	-6.08	0.001338	-1	244065_at	CNTNAP3B
-0.664	-6.07	0.001365	-1	203476_at	TPBG
-0.506	-6.06	0.001388	-1	240983_s_at	CARS
-0.426	-6.05	0.001392	-1	202467_s_at	COPS2
-2.399	-6.04	0.001392	-1	206858_s_at	HOXC6
-1.472	-6.03	0.001399	-1	213844_at	HOXA5
-0.568	-6.03	0.001401	-1	228575_at	IL20RB
-0.939	-6.01	0.001438	-1	214724_at	DIXDC1
-0.531	-5.97	0.001541	-1	206463_s_at	DHRS2
-0.693	-5.97	0.001541	-1	231094_s_at	MTHFD1L
-0.646	-5.95	0.001592	-1	212048_s_at	YARS
-0.207	-5.89	0.001766	-1	1562378_s_at	PROM2
-0.541	-5.89	0.001777	-1	208768_x_at	RPL22
-0.161	-5.87	0.001833	-1	244746_at	SEMA6D
-0.915	-5.78	0.002203	-1	230563_at	RASGEF1A
-0.49	-5.75	0.002286	-1	208854_s_at	STK24
-0.362	-5.75	0.002293	-1	230326_s_at	C11orf73
-0.622	-5.75	0.002305	-1	213716_s_at	SECTM1
-0.356	-5.73	0.002374	-1	200005_at	EIF3D
-0.206	-5.73	0.002374	-1	210807_s_at	SLC16A7
-0.684	-5.7	0.002511	-1	204654_s_at	TFAP2A
-0.702	-5.7	0.002511	-1	225955_at	METRNL
-0.783	-5.69	0.002544	-1	227452_at	NA
-0.56	-5.68	0.002564	-1	210357_s_at	SMOX
-0.341	-5.68	0.002579	-1	1558342_x_at	DIXDC1
-0.157	-5.67	0.002579	-1	1566989_at	ARID1B
-0.567	-5.67	0.002579	-1	202236_s_at	SLC16A1
-0.497	-5.67	0.002579	-1	218556_at	ORMDL2
-0.303	-5.66	0.002626	-1	1568742_at	NA
-1.218	-5.66	0.002626	-1	205730_s_at	ABLIM3
-1.306	-5.62	0.002823	-1	214079_at	DHRS2
-0.707	-5.61	0.002848	-1	200082_s_at	RPS7
-0.457	-5.57	0.003123	-1	221490_at	UBAP1
-1.082	-5.57	0.003126	-1	202766_s_at	FBN1
-0.479	-5.56	0.003129	-1	214167_s_at	NA
-0.521	-5.56	0.00315	-1	209668_x_at	CES2
-0.305	-5.54	0.003279	-1	218208_at	PQLC1
-0.663	-5.52	0.003375	-1	201324_at	EMP1
-0.282	-5.49	0.003528	-1	238987_at	B4GALT1
-0.407	-5.43	0.003855	-1	209213_at	CBR1
-0.515	-5.43	0.003855	-1	211710_x_at	RPL4

Coef.ft.n...ov.n	t.ft.n...ov.n	p.value.adj.ft.n...ov.n	Res.ft.n...ov.n	ID	gene.symbols
-0.494	-5.41	0.004072	-1	220960_x_at	RPL22
-0.448	-5.41	0.004072	-1	231559_at	NNMT
-0.787	-5.4	0.004116	-1	204900_x_at	SAP30
-0.429	-5.38	0.00428	-1	208726_s_at	EIF2S2
-0.334	-5.38	0.00428	-1	222670_s_at	MAFB
-0.995	-5.34	0.004603	-1	225582_at	ITPRIP
-0.371	-5.28	0.005091	-1	209409_at	GRB10
-0.392	-5.26	0.005255	-1	201145_at	HAX1
-1.28	-5.26	0.005255	-1	220892_s_at	PSAT1
-0.984	-5.26	0.005255	-1	239761_at	GCNT1
-0.744	-5.26	0.005307	-1	225520_at	MTHFD1L
-0.398	-5.25	0.005318	-1	202402_s_at	CARS
-0.39	-5.25	0.00534	-1	224463_s_at	C11orf70
-0.206	-5.25	0.005342	-1	207449_s_at	POFUT2
-0.433	-5.24	0.005401	-1	228234_at	NA
-0.328	-5.24	0.005403	-1	214442_s_at	PIAS2
-0.257	-5.22	0.00555	-1	200890_s_at	SSR1
-0.555	-5.22	0.00555	-1	212295_s_at	SLC7A1
-0.497	-5.22	0.00555	-1	214643_x_at	BIN1
-0.36	-5.22	0.00555	-1	220980_s_at	ADPGK
-0.483	-5.22	0.00555	-1	223796_at	CNTNAP3
-0.337	-5.21	0.00559	-1	204374_s_at	GALK1
-1.263	-5.19	0.00576	-1	202238_s_at	NNMT
-0.78	-5.15	0.006167	-1	205505_at	GCNT1
-0.146	-5.15	0.006167	-1	232236_at	NA
-2.492	-5.14	0.00628	-1	209821_at	IL33
-0.508	-5.13	0.00636	-1	201154_x_at	RPL4
-0.415	-5.12	0.006463	-1	227406_at	LOC100129387
-1.446	-5.11	0.006518	-1	212190_at	SERPINE2
-0.432	-5.1	0.006619	-1	212971_at	CARS
-0.307	-5.1	0.006621	-1	201994_at	MORF4L2
-1.218	-5.1	0.006621	-1	219049_at	CSGALNACT1
-0.434	-5.09	0.006653	-1	202142_at	COPS8
-0.488	-5.08	0.006798	-1	222644_s_at	GLT25D1
-1.175	-5.07	0.006989	-1	202237_at	NNMT
-0.711	-5.06	0.007025	-1	231698_at	NA
-0.946	-5.06	0.00705	-1	204653_at	TFAP2A
-0.531	-5.06	0.00705	-1	229432_at	NAGS
-0.437	-5.04	0.0073	-1	228083_at	CACNA2D4
-1.487	-5.04	0.00731	-1	202388_at	RGS2
-0.497	-5.03	0.00735	-1	216336_x_at	NA
-0.256	-5.03	0.00735	-1	227421_at	C21orf57
-0.454	-5.03	0.007364	-1	1564027_a_at	FAM115C
-0.387	-5	0.007717	-1	200022_at	RPL18
-0.359	-4.99	0.007832	-1	201033_x_at	RPLP0

Coef.ft.n...ov.n	t.ft.n...ov.n	p.value.adj.ft.n...ov.n	Res.ft.n...ov.n	ID	gene.symbols
-0.721	-4.98	0.007937	-1	209681_at	SLC19A2
-0.312	-4.98	0.007967	-1	216483_s_at	C19orf10
-0.328	-4.98	0.008056	-1	219573_at	LRRC16A
-0.226	-4.97	0.008149	-1	209371_s_at	SH3BP2
-0.544	-4.97	0.008153	-1	1553768_a_at	DCBLD1
-1.063	-4.93	0.008678	-1	204389_at	MAOA
-0.614	-4.92	0.008817	-1	209448_at	HTATIP2
-0.687	-4.92	0.008887	-1	205668_at	LY75
-0.359	-4.92	0.008919	-1	213588_x_at	RPL14
-0.576	-4.91	0.008938	-1	218497_s_at	RNASEH1
-0.585	-4.91	0.008946	-1	201325_s_at	EMP1
-0.398	-4.91	0.009021	-1	202123_s_at	ABL1
-0.59	-4.9	0.009073	-1	218559_s_at	MAFB
-0.29	-4.9	0.009159	-1	203839_s_at	TNK2
-0.373	-4.88	0.009428	-1	211073_x_at	RPL3
-0.186	-4.87	0.009568	-1	223857_x_at	TMEM85
-0.541	-4.87	0.009729	-1	1557918_s_at	SLC16A1
-0.36	-4.86	0.009738	-1	201217_x_at	RPL3
-1.475	-4.86	0.009738	-1	219694_at	FAM105A
-0.504	-4.86	0.009863	-1	204744_s_at	IARS
-0.797	-4.84	0.010223	-1	208693_s_at	GARS
-0.829	-4.83	0.010343	-1	234725_s_at	SEMA4B
-0.475	-4.83	0.010357	-1	223151_at	DCUN1D5
-0.395	-4.81	0.010708	-1	212246_at	MCFD2
-1.128	-4.81	0.01087	-1	1553972_a_at	CBS
-0.944	-4.8	0.010874	-1	221423_s_at	YIPF5
-0.593	-4.79	0.011041	-1	222646_s_at	ERO1L
-0.359	-4.79	0.011042	-1	211972_x_at	RPLP0
-1.114	-4.78	0.011202	-1	219682_s_at	TBX3
-0.268	-4.75	0.012002	-1	206621_s_at	EIF4H
-0.421	-4.75	0.012002	-1	209537_at	EXTL2
-0.284	-4.75	0.012002	-1	221739_at	C19orf10
-0.704	-4.75	0.012002	-1	222620_s_at	DNAJC1
-0.961	-4.74	0.01215	-1	212816_s_at	CBS
-0.464	-4.74	0.012163	-1	222768_s_at	TRMT6
-1.807	-4.73	0.012358	-1	201438_at	COL6A3
-0.343	-4.73	0.012358	-1	230370_x_at	STYXL1
-0.55	-4.71	0.012676	-1	201628_s_at	RRAGA
-0.434	-4.71	0.012831	-1	204119_s_at	ADK
-0.34	-4.71	0.012831	-1	211487_x_at	RPS17
-0.474	-4.71	0.012831	-1	226609_at	DCBLD1
-0.543	-4.7	0.012869	-1	217165_x_at	MT1F
-0.579	-4.7	0.012976	-1	231936_at	HOXC9
-1.228	-4.7	0.012976	-1	244650_at	FAM105A
-0.376	-4.69	0.013194	-1	218099_at	TEX2

Coef.ft.n...ov.n	t.ft.n...ov.n	p.value.adj.ft.n...ov.n	Res.ft.n...ov.n	ID	gene.symbols
-0.328	-4.69	0.013194	-1	218945_at	C16orf68
-0.329	-4.67	0.013603	-1	209743_s_at	ITCH
-0.847	-4.67	0.013651	-1	229576_s_at	TBX3
-0.369	-4.66	0.013895	-1	201665_x_at	RPS17
-0.844	-4.66	0.013895	-1	205480_s_at	UGP2
-0.955	-4.65	0.013965	-1	204388_s_at	MAOA
-0.388	-4.65	0.014069	-1	201866_s_at	NR3C1
-0.466	-4.65	0.014072	-1	201475_x_at	MARS
-0.773	-4.65	0.014072	-1	206631_at	PTGER2
-0.432	-4.65	0.014072	-1	211666_x_at	RPL3
-0.32	-4.63	0.014378	-1	213892_s_at	APRT
-0.737	-4.62	0.014578	-1	206090_s_at	DISC1
-0.209	-4.62	0.014589	-1	209495_at	CEP250
-0.325	-4.62	0.014589	-1	219215_s_at	SLC39A4
-0.434	-4.62	0.014738	-1	200036_s_at	RPL10A
-0.351	-4.61	0.014799	-1	211456_x_at	MT1P2
-0.34	-4.61	0.014853	-1	211720_x_at	RPLP0
-0.36	-4.6	0.014975	-1	208856_x_at	RPLP0
-0.951	-4.6	0.015124	-1	1557905_s_at	CD44
-0.493	-4.6	0.015124	-1	238575_at	OSBPL6
-0.371	-4.59	0.015441	-1	235419_at	NA
-0.383	-4.58	0.015526	-1	203111_s_at	PTK2B
-0.706	-4.58	0.015715	-1	204326_x_at	MT1X
-0.93	-4.57	0.015715	-1	244025_at	NA
-1.459	-4.57	0.015719	-1	201842_s_at	EFEMP1
-0.812	-4.57	0.015719	-1	205421_at	SLC22A3
-0.369	-4.56	0.015967	-1	207788_s_at	SORBS3
-0.289	-4.56	0.015967	-1	233982_x_at	STYXL1
-0.181	-4.56	0.016092	-1	229565_x_at	NA
-0.418	-4.56	0.016167	-1	202141_s_at	COPS8
-0.289	-4.55	0.016347	-1	1554553_s_at	YIF1B
-0.483	-4.54	0.016347	-1	1555680_a_at	SMOX
-0.328	-4.55	0.016347	-1	203516_at	SNTA1
-0.358	-4.55	0.016347	-1	212039_x_at	RPL3
-0.487	-4.54	0.016373	-1	200822_x_at	TPI1
-0.373	-4.54	0.016497	-1	208855_s_at	STK24
-0.542	-4.54	0.016521	-1	221827_at	RBCK1
-0.304	-4.53	0.016568	-1	214317_x_at	RPS9
-0.514	-4.53	0.016568	-1	214954_at	SUSD5
-0.46	-4.53	0.016568	-1	218409_s_at	DNAJC1
-0.285	-4.53	0.016568	-1	222978_at	SURF4
-0.131	-4.5	0.017518	-1	215562_at	TTC39A
-0.153	-4.5	0.017673	-1	207360_s_at	NTSR1
-0.362	-4.49	0.017788	-1	200654_at	P4HB
-0.34	-4.49	0.017788	-1	212578_x_at	RPS17

Coef.ft.n...ov.n	t.ft.n...ov.n	p.value.adj.ft.n...ov.n	Res.ft.n...ov.n	ID	gene.symbols
-0.469	-4.49	0.017946	-1	215416_s_at	STOML2
-0.582	-4.48	0.018043	-1	213941_x_at	RPS7
-0.869	-4.48	0.018085	-1	224949_at	YIPF5
-0.4	-4.48	0.018133	-1	201254_x_at	RPS6
-0.343	-4.48	0.018165	-1	232353_s_at	STYXL1
-0.383	-4.47	0.018459	-1	200937_s_at	RPL5
-0.511	-4.46	0.018538	-1	213419_at	APBB2
-0.229	-4.46	0.018555	-1	239236_at	NA
-0.468	-4.46	0.018692	-1	226506_at	THSD4
-0.407	-4.44	0.019079	-1	212859_x_at	MT1E
-0.207	-4.43	0.01928	-1	1555557_a_at	TNK2
-0.504	-4.43	0.019346	-1	208629_s_at	HADHA
-1.405	-4.43	0.019494	-1	228501_at	GALNTL2
-0.825	-4.43	0.019505	-1	226136_at	GLIPR1
-0.619	-4.42	0.019621	-1	207480_s_at	MEIS2
-0.348	-4.42	0.019638	-1	219488_at	A4GALT
-0.328	-4.42	0.019657	-1	201239_s_at	NA
-0.243	-4.42	0.019657	-1	234936_s_at	CC2D2A
-0.249	-4.41	0.0198	-1	218484_at	NDUFA4L2
-0.622	-4.41	0.0198	-1	222621_at	DNAJC1
-0.272	-4.41	0.019972	-1	1552690_a_at	CACNA2D4
-0.593	-4.41	0.019972	-1	203167_at	TIMP2
-0.342	-4.41	0.019972	-1	218216_x_at	ARL6IP4
-0.477	-4.4	0.020347	-1	218843_at	FNDC4
-1.444	-4.39	0.02069	-1	201843_s_at	EFEMP1
-0.27	-4.39	0.020698	-1	201622_at	SND1
-0.403	-4.38	0.020753	-1	212018_s_at	RSL1D1
-0.351	-4.38	0.020756	-1	209137_s_at	USP10
-0.19	-4.38	0.02079	-1	203727_at	SKIV2L
-0.537	-4.38	0.020804	-1	217915_s_at	RSL24D1
-0.502	-4.38	0.020821	-1	208631_s_at	HADHA
-0.264	-4.38	0.020821	-1	208887_at	EIF3G
-0.409	-4.38	0.02084	-1	203484_at	SEC61G
-0.373	-4.37	0.02089	-1	201562_s_at	SORD
-0.979	-4.37	0.020939	-1	214085_x_at	GLIPR1
-0.539	-4.37	0.020957	-1	225136_at	PLEKHA2
-0.236	-4.37	0.02098	-1	202632_at	NA
-0.741	-4.37	0.02098	-1	221539_at	EIF4EBP1
-0.344	-4.36	0.021055	-1	221691_x_at	NPM1
-0.182	-4.36	0.02115	-1	1562446_at	ZNF391
-0.341	-4.35	0.021392	-1	203107_x_at	RPS2
-0.961	-4.35	0.021501	-1	204490_s_at	CD44
-1.141	-4.34	0.021777	-1	201397_at	PHGDH
-0.787	-4.34	0.021777	-1	202619_s_at	PLOD2
-0.289	-4.34	0.021814	-1	218321_x_at	STYXL1

Coef.ft.n...ov.n	t.ft.n...ov.n	p.value.adj.ft.n...ov.n	Res.ft.n...ov.n	ID	gene.symbols
-0.5	-4.33	0.022293	-1	210951_x_at	RAB27A
-0.331	-4.32	0.022491	-1	1552914_a_at	CD276
-0.765	-4.32	0.022491	-1	210916_s_at	CD44
-0.48	-4.32	0.022491	-1	223416_at	SF3B14
-0.283	-4.32	0.022491	-1	228890_at	ATOH8
-0.714	-4.32	0.022491	-1	231148_at	IGFL2
-1.177	-4.32	0.022549	-1	223062_s_at	PSAT1
-0.273	-4.31	0.022549	-1	224455_s_at	ADPGK
-1.793	-4.31	0.022601	-1	236361_at	GALNTL2
-0.301	-4.31	0.022691	-1	201600_at	PHB2
-0.247	-4.31	0.022764	-1	200017_at	RPS27A
-0.461	-4.31	0.022764	-1	217140_s_at	VDAC1
-0.315	-4.29	0.023591	-1	224334_s_at	NA
-0.383	-4.29	0.023591	-1	228222_at	NA
-0.715	-4.28	0.023644	-1	204541_at	SEC14L2
-0.466	-4.28	0.023717	-1	200089_s_at	RPL4
-0.269	-4.28	0.023717	-1	203219_s_at	APRT
-0.515	-4.28	0.023717	-1	218258_at	POLR1D
-0.259	-4.28	0.023782	-1	208628_s_at	YBX1
-0.52	-4.28	0.023782	-1	217504_at	ABCA6
-0.341	-4.27	0.023939	-1	215963_x_at	NA
-0.162	-4.26	0.024203	-1	204553_x_at	INPP4A
-0.947	-4.27	0.024203	-1	206377_at	FOXF2
-0.839	-4.27	0.024203	-1	225544_at	TBX3
-0.253	-4.26	0.024252	-1	211939_x_at	BTF3
-0.595	-4.26	0.024264	-1	201060_x_at	STOM
-0.438	-4.25	0.024641	-1	204745_x_at	MT1G
-0.419	-4.24	0.025167	-1	200081_s_at	RPS6
-0.771	-4.23	0.025759	-1	224953_at	YIPF5
-0.342	-4.23	0.025889	-1	201914_s_at	SEC63
-0.275	-4.23	0.025889	-1	203256_at	CDH3
-0.491	-4.23	0.025889	-1	224601_at	NA
-0.371	-4.22	0.025943	-1	200968_s_at	PPIB
-0.376	-4.22	0.025943	-1	217747_s_at	RPS9
-0.241	-4.23	0.025943	-1	236616_at	NA
-0.273	-4.22	0.025953	-1	200018_at	RPS13
-0.422	-4.22	0.025953	-1	218473_s_at	GLT25D1
-0.307	-4.22	0.02603	-1	200079_s_at	KARS
-0.16	-4.22	0.02603	-1	216114_at	NCKIPSD
-1.406	-4.21	0.026297	-1	200878_at	EPAS1
-0.99	-4.21	0.026297	-1	209835_x_at	CD44
-0.282	-4.21	0.026363	-1	225190_x_at	RPL35A
-0.436	-4.21	0.026475	-1	200725_x_at	RPL10
-0.48	-4.2	0.026594	-1	200715_x_at	RPL13A
-0.281	-4.2	0.026781	-1	228164_at	AP4E1

Coef.ft.n...ov.n	t.ft.n...ov.n	p.value.adj.ft.n...ov.n	Res.ft.n...ov.n	ID	gene.symbols
-0.41	-4.19	0.027308	-1	200656_s_at	P4HB
-0.334	-4.19	0.027324	-1	208627_s_at	YBX1
-0.313	-4.19	0.027324	-1	208967_s_at	AK2
-0.327	-4.18	0.027456	-1	203113_s_at	EEF1D
-0.846	-4.18	0.027456	-1	209276_s_at	GLRX
-0.543	-4.18	0.027498	-1	213629_x_at	MT1F
-0.529	-4.18	0.027602	-1	223805_at	OSBPL6
-0.234	-4.17	0.027843	-1	200029_at	RPL19
-0.793	-4.17	0.027891	-1	204221_x_at	GLIPR1
-0.35	-4.17	0.028256	-1	208510_s_at	PPARG
-0.573	-4.16	0.028256	-1	212290_at	SLC7A1
-0.182	-4.16	0.028256	-1	38340_at	NA
-0.216	-4.16	0.028593	-1	211592_s_at	CACNA1C
-0.961	-4.15	0.029295	-1	218631_at	AVPI1
-0.304	-4.15	0.029347	-1	200735_x_at	NACA
-0.492	-4.15	0.029347	-1	206566_at	SLC7A1
-0.246	-4.14	0.02942	-1	226257_x_at	MRPS22
-0.359	-4.14	0.02942	-1	228822_s_at	USP16
-0.321	-4.14	0.029427	-1	217466_x_at	RPS2
-0.339	-4.14	0.029617	-1	227970_at	GPR157
-0.535	-4.13	0.029983	-1	210186_s_at	FKBP1A
-0.307	-4.12	0.030186	-1	204857_at	MAD1L1
-0.963	-4.12	0.030186	-1	212014_x_at	CD44
-0.318	-4.12	0.030186	-1	220597_s_at	ARL6IP4
-0.185	-4.13	0.030186	-1	230186_at	TMEM136
-0.339	-4.12	0.030518	-1	212292_at	SLC7A1
-0.144	-4.12	0.030689	-1	232180_at	UGP2
-0.288	-4.11	0.030721	-1	200926_at	RPS23
-0.419	-4.11	0.031044	-1	228421_s_at	EFEMP1
-0.347	-4.11	0.031074	-1	223018_at	NOB1
-0.309	-4.11	0.031074	-1	234873_x_at	RPL7A
-0.472	-4.11	0.031083	-1	212085_at	SLC25A6
-0.444	-4.1	0.031108	-1	226311_at	NA
-0.463	-4.1	0.031154	-1	202148_s_at	PYCR1
-0.142	-4.1	0.031431	-1	214015_at	SOCS7
-0.405	-4.1	0.031563	-1	226025_at	ANKRD28
-0.462	-4.09	0.031563	-1	230793_at	LRRC16A
-0.312	-4.09	0.031776	-1	209360_s_at	RUNX1
-0.241	-4.09	0.031932	-1	223664_x_at	BCL2L13
-0.213	-4.09	0.031971	-1	203838_s_at	TNK2
-0.354	-4.08	0.032051	-1	224321_at	TMEFF2
-0.333	-4.08	0.032172	-1	201251_at	PKM2
-0.445	-4.08	0.032172	-1	213011_s_at	TPI1
-0.472	-4.08	0.032329	-1	204053_x_at	PTEN
-0.315	-4.08	0.032382	-1	229563_s_at	RPL10A

Coef.ft.n...ov.n	t.ft.n...ov.n	p.value.adj.ft.n...ov.n	Res.ft.n...ov.n	ID	gene.symbols
-0.185	-4.08	0.032415	-1	218672_at	SCNM1
-1.164	-4.08	0.032534	-1	225420_at	GPAM
-0.188	-4.05	0.034313	-1	228348_at	LINS1
-0.232	-4.05	0.034349	-1	206221_at	RASA3
-0.122	-4.05	0.034349	-1	211395_x_at	FCGR2C
-0.305	-4.04	0.034632	-1	202591_s_at	SSBP1
-0.349	-4.04	0.034632	-1	213801_x_at	RPSA
-0.179	-4.04	0.034632	-1	226754_at	ZNF251
-0.174	-4.04	0.034719	-1	223684_s_at	SMUG1
-0.209	-4.03	0.035	-1	206016_at	CCDC22
-0.272	-4.03	0.035	-1	211542_x_at	RPS10
-0.28	-4.03	0.035234	-1	241198_s_at	C11orf70
-0.44	-4.03	0.035314	-1	208581_x_at	MT1X
-0.144	-4.03	0.035314	-1	215670_s_at	SCAND2
-0.78	-4.03	0.035331	-1	226614_s_at	FAM167A
-0.429	-4.02	0.0354	-1	209900_s_at	SLC16A1
-0.268	-4.02	0.0356	-1	200936_at	RPL8
-0.15	-4.02	0.035696	-1	234746_at	NA
-0.362	-4.01	0.036057	-1	209962_at	EPOR
-0.304	-4.01	0.036057	-1	230003_at	NA
-0.533	-4.01	0.036232	-1	216521_s_at	BRCC3
-1.353	-4.01	0.036261	-1	1555271_a_at	TERT
-0.193	-4.01	0.036369	-1	200619_at	SF3B2
-0.475	-4.01	0.036369	-1	209514_s_at	RAB27A
-0.259	-4	0.036574	-1	203212_s_at	MTMR2
-0.186	-4	0.036574	-1	214057_at	MCL1
-0.257	-4	0.036735	-1	200095_x_at	RPS10
-0.322	-4	0.036735	-1	200967_at	PPIB
-0.202	-4	0.036735	-1	217559_at	RPL10L
-0.24	-4	0.036762	-1	201330_at	RARS
-0.291	-3.99	0.036941	-1	203732_at	TRIP4
-0.264	-3.99	0.036997	-1	225841_at	C1orf59
-0.273	-3.99	0.037495	-1	201716_at	SNX1
-0.345	-3.98	0.037824	-1	217677_at	PLEKHA2
-0.261	-3.98	0.037843	-1	200063_s_at	NPM1
-0.349	-3.98	0.037858	-1	213076_at	ITPKC
-0.105	-3.97	0.037992	-1	207151_at	ADCYAP1R1
-0.359	-3.97	0.038115	-1	233952_s_at	ZNF295
-0.195	-3.97	0.038179	-1	1569202_x_at	NA
-0.68	-3.97	0.038179	-1	202862_at	FAH
-0.281	-3.97	0.038179	-1	208635_x_at	NACA
-0.193	-3.97	0.03822	-1	205315_s_at	SNTB2
-1.611	-3.96	0.03841	-1	203083_at	THBS2
-0.393	-3.96	0.038542	-1	200670_at	XBP1
-0.957	-3.96	0.038542	-1	206295_at	IL18

Coef.ft.n...ov.n	t.ft.n...ov.n	p.value.adj.ft.n...ov.n	Res.ft.n...ov.n	ID	gene.symbols
-0.914	-3.96	0.038542	-1	212741_at	MAOA
-0.534	-3.96	0.038542	-1	222465_at	RSL24D1
-0.324	-3.96	0.038714	-1	217740_x_at	RPL7A
-0.393	-3.95	0.038874	-1	203110_at	PTK2B
-0.323	-3.95	0.038874	-1	225363_at	PTEN
-0.601	-3.95	0.038885	-1	200769_s_at	MAT2A
-0.624	-3.95	0.038885	-1	201563_at	SORD
-0.231	-3.95	0.039006	-1	203040_s_at	HMBS
-0.285	-3.95	0.039006	-1	228413_s_at	SFRP1
-0.281	-3.95	0.039081	-1	200889_s_at	SSR1
-0.362	-3.95	0.039081	-1	200898_s_at	MGEA5
-0.286	-3.95	0.039081	-1	217841_s_at	PPME1
-0.29	-3.95	0.039101	-1	220887_at	C14orf162
-0.438	-3.94	0.039153	-1	201306_s_at	ANP32B
-0.553	-3.94	0.039199	-1	208978_at	CRIP2
-0.343	-3.94	0.039347	-1	215794_x_at	GLUD2
-0.329	-3.94	0.039727	-1	202214_s_at	CUL4B
-0.24	-3.93	0.039882	-1	202255_s_at	SIPA1L1
-0.683	-3.93	0.039938	-1	224856_at	FKBP5
-0.336	-3.93	0.039959	-1	202030_at	BCKDK
-0.366	-3.93	0.039959	-1	212433_x_at	RPS2
-0.272	-3.93	0.039959	-1	212769_at	TLE3
-0.204	-3.93	0.039959	-1	218056_at	BFAR
-1.32	-3.93	0.039988	-1	224657_at	ERRFI1
-0.615	-3.93	0.040051	-1	206662_at	GLRX
-0.333	-3.93	0.040135	-1	217094_s_at	ITCH
-1.107	-3.93	0.040156	-1	212488_at	COL5A1
-0.106	-3.92	0.040322	-1	221385_s_at	FFAR3
-0.282	-3.92	0.040445	-1	212396_s_at	KIAA0090
-0.5	-3.92	0.040534	-1	211937_at	EIF4B
-1.156	-3.92	0.040534	-1	218211_s_at	MLPH
-0.201	-3.92	0.040668	-1	207118_s_at	NA
-0.528	-3.91	0.040993	-1	223306_at	EBPL
-0.105	-3.91	0.041065	-1	221863_at	MIER2
-0.187	-3.91	0.041162	-1	202140_s_at	CLK3
-0.566	-3.91	0.041201	-1	200693_at	YWHAQ
-0.238	-3.91	0.041201	-1	208517_x_at	BTF3
-0.311	-3.91	0.041201	-1	208680_at	PRDX1
-0.309	-3.91	0.041225	-1	240076_at	NA
-0.435	-3.9	0.041656	-1	201231_s_at	ENO1
-0.26	-3.9	0.041656	-1	202247_s_at	MTA1
-0.274	-3.9	0.041656	-1	212734_x_at	RPL13
-0.221	-3.9	0.041656	-1	224951_at	LASS5
-0.188	-3.9	0.041678	-1	228440_at	NA
-0.358	-3.89	0.041938	-1	200946_x_at	GLUD1

Coef.ft.n...ov.n	t.ft.n...ov.n	p.value.adj.ft.n...ov.n	Res.ft.n...ov.n	ID	gene.symbols
-0.387	-3.89	0.042027	-1	204156_at	SIK3
-0.235	-3.89	0.042053	-1	209565_at	RNF113A
-0.161	-3.89	0.042057	-1	227895_at	FAM120B
-0.267	-3.89	0.042267	-1	204824_at	ENDOG
-0.309	-3.89	0.042323	-1	205609_at	ANGPT1
-0.141	-3.88	0.0424	-1	211326_x_at	HFE
-0.332	-3.88	0.042692	-1	233364_s_at	NA
-0.153	-3.88	0.042742	-1	204833_at	ATG12
-0.419	-3.87	0.042884	-1	201892_s_at	IMPDH2
-0.316	-3.88	0.042884	-1	213080_x_at	RPL5
-0.446	-3.88	0.042884	-1	221967_at	NXPH4
-0.694	-3.87	0.042925	-1	209580_s_at	MBD4
-0.317	-3.87	0.042934	-1	218021_at	NA
-0.432	-3.87	0.042944	-1	209228_x_at	TUSC3
-0.249	-3.87	0.043148	-1	221494_x_at	EIF3K
-0.238	-3.87	0.043149	-1	218220_at	C12orf10
-0.817	-3.87	0.043223	-1	202847_at	PCK2
-0.405	-3.87	0.043223	-1	224663_s_at	CFL2
-0.395	-3.86	0.043533	-1	202670_at	MAP2K1
-0.477	-3.86	0.043783	-1	203336_s_at	ITGB1BP1
-0.275	-3.86	0.043857	-1	232689_at	LOC284561
-0.801	-3.86	0.044088	-1	224934_at	YIPF5
-0.239	-3.85	0.04415	-1	221039_s_at	ASAP1
-0.418	-3.85	0.044503	-1	210257_x_at	CUL4B
-0.121	-3.85	0.04462	-1	239085_at	JDP2
-0.313	-3.85	0.044621	-1	212191_x_at	RPL13
-1.427	-3.85	0.044647	-1	221921_s_at	CADM3
-0.12	-3.84	0.044829	-1	1553872_at	C17orf103
-0.35	-3.84	0.044829	-1	209134_s_at	RPS6
-0.523	-3.84	0.044975	-1	222835_at	THSD4
-0.123	-3.84	0.044975	-1	240656_at	NA
-0.395	-3.84	0.045021	-1	212038_s_at	VDAC1
-0.534	-3.84	0.045021	-1	226982_at	ELL2
-0.528	-3.84	0.045066	-1	200768_s_at	MAT2A
-0.174	-3.83	0.0457	-1	239492_at	SEC14L4
-1.432	-3.83	0.04578	-1	206432_at	HAS2
-0.335	-3.82	0.045948	-1	217891_at	C16orf58
-0.136	-3.82	0.046007	-1	1555820_a_at	MKS1
-0.213	-3.82	0.046007	-1	203133_at	SEC61B
-0.411	-3.82	0.046007	-1	213982_s_at	RABGAP1L
-1.152	-3.82	0.046007	-1	230746_s_at	NA
-0.129	-3.82	0.046089	-1	1560156_at	NA
-0.501	-3.82	0.046249	-1	229158_at	WNK4
-0.441	-3.82	0.046403	-1	222728_s_at	TAF1D
-0.388	-3.82	0.046426	-1	226504_at	FAM109B

Coef.ft.n...ov.n	t.ft.n...ov.n	p.value.adj.ft.n...ov.n	Res.ft.n...ov.n	ID	gene.symbols
-0.172	-3.81	0.046443	-1	207795_s_at	KLRD1
-1.036	-3.81	0.046447	-1	204222_s_at	GLIPR1
-1.232	-3.81	0.046447	-1	205990_s_at	WNT5A
-0.982	-3.81	0.046447	-1	225424_at	GPAM
-0.227	-3.81	0.046771	-1	218415_at	VPS33B
-0.531	-3.81	0.046771	-1	226726_at	MBOAT2
-0.318	-3.8	0.047057	-1	200899_s_at	MGEA5
-0.375	-3.8	0.047082	-1	204429_s_at	SLC2A5
-0.27	-3.8	0.047171	-1	218315_s_at	CDK5RAP1
-0.293	-3.8	0.047171	-1	222616_s_at	USP16
-0.399	-3.8	0.047279	-1	208692_at	RPS3
-0.623	-3.8	0.04742	-1	201061_s_at	STOM
-0.407	-3.8	0.04742	-1	218189_s_at	NANS
-0.751	-3.79	0.047517	-1	1555788_a_at	TRIB3
-0.715	-3.79	0.047775	-1	204879_at	PDPN
-0.489	-3.79	0.04781	-1	1564494_s_at	P4HB
-0.277	-3.79	0.047923	-1	201871_s_at	UBXN1
-0.235	-3.79	0.047969	-1	204031_s_at	PCBP2
-0.876	-3.79	0.048069	-1	226142_at	GLIPR1
-0.186	-3.78	0.048332	-1	218075_at	AAAS
-0.247	-3.78	0.048332	-1	224479_s_at	MRPL45
-0.339	-3.78	0.048494	-1	212582_at	OSBPL8
-0.225	-3.78	0.048687	-1	225403_at	C9orf23
-0.114	-3.78	0.048722	-1	222041_at	NA
-0.482	-3.78	0.048731	-1	225489_at	TMEM18
-0.255	-3.77	0.049251	-1	201257_x_at	RPS3A
-0.225	-3.77	0.049269	-1	396_f_at	EPOR
-0.281	-3.77	0.049386	-1	222977_at	SURF4
-0.132	-3.76	0.049855	-1	214585_s_at	VPS52
-0.2	-3.76	0.049872	-1	228059_x_at	NA
