# Supplementary material for: Gene Expression Signature of Normal Cell-of-Origin Predicts Ovarian Tumor Outcomes
Source: PLoS One. 2013 Nov 26;8(11):e80314. doi: 10.1371/journal.pone.0080314 (PMC3841174; doi:10.1371/journal.pone.0080314)
Supplement: File S1 — Combined Supporting Information file including the Supplementary Methods and the specified Supplementary Tables and Figures. (DOCX) [file pone.0080314.s005.docx]

**Merritt *et al*. Supplementary File S1**

**SUPPLEMENTARY METHODS**

**Cell Culture Medium**

Previously we described several versions of a novel chemically-defined cell culture medium (WIT) that can support long-term growth of normal and transformed human breast cells without undefined components such as serum, feeder-layers, tissue extracts or pharmacological reagents [1]. In WIT medium normal human breast epithelial cells reached 70 population doublings during six months of continuous culture, a nearly 10^21^-fold expansion of cell number, suggesting that they were able maintain a healthy metabolism without any additives or serum.

Interestingly, the WIT medium developed for normal human breast cells did not support the growth of normal ovarian or fallopian tube cells. Hence, we began making modifications to the WIT medium to optimize it to culture normal ovarian epithelial and fallopian tube epithelial cells. After trying out many different modifications over several years, we were able to culture normal ovarian epithelial and fallopian tube epithelial cells by adjusting the final concentrations of four components in WIT medium: EGF (0.01 ug/mL, E9644, Sigma-Aldrich, St. Louis, MO), Insulin (20 ug/mL, I0516, Sigma-Aldrich), Hydrocortisone (0.5 ug/mL, H0888, Sigma-Aldrich), 25ng/mL Cholera Toxin (227035, Calbiochem, EMD Millipore, Billerica, MA).

We also reasoned that low amounts of serum may be required to mimic the physiologic environment of normal ovary and fallopian tube. The normal human breast cells, like most epithelium, are never in direct contact with blood or serum under physiologic conditions. Accordingly, the medium we developed for normal breast cells was completely devoid of serum in order to mimic physiologic conditions. In contrast, the ovaries and fallopian tubes are directly in contact with normal peritoneal fluid, which contains a physiologic serum protein concentration that can be as high as 50% of the circulating blood [2]. Consistent with these observations, serum inhibited normal breast epithelial cell growth; however, addition of 0.5- 1% heat inactivated fetal bovine serum (HyClone, Thermo Fisher Scientific, Waltham, MA) increased the growth of normal ovarian epithelial and fallopian tube epithelial cells. The medium that combines these modifications; Cholera Toxin (25ng/mL), EGF (0.01 ug/mL), Insulin (20 ug/mL), Hydrocortisone (0.5 ug/mL) and heat inactivated fetal bovine serum (0.5 - 1%) is referred as WIT-fo.

A number of different media have been previously used to culture ovarian epithelial [3,4,5] and fallopian tube epithelial cells [6,7,8]. These media are generally comprised of 40 to 55 ingredients supplemented with 10-15% serum; MCDB 105/Medium 199, DMEM, DMEM/ Ham’s F12, RPMI-1640. In contrast WIT-fo medium is composed of >80 components and is supplemented with 0.5% serum. Testing combinations of 30 to 35 WIT-fo components which are usually absent in standard media in order to determine which components are absolutely necessary for the difference in the performance of these media would require examining more than 1.1 x 10^9^ different conditions, which is well beyond the capability of an academic laboratory. Thus, at this point it is difficult to pinpoint the exact components responsible for the ability of WIT-fo to support normal ovarian epithelial and fallopian tube epithelial cells because of the large number of differences between the WIT and the standard cell culture media.

**Tissue collection**

All study procedures were approved by the Internal Review Board at the Brigham and Women’s Hospital to collect discarded tissues. The study protocol allowed limited access to clinical information to exclude women from the study if they had an increased familial risk for ovarian cancer or those currently taking medications that could modify their ovaries or fallopian tubes.

Collecting surface scrapings during surgery was most successful to establish long term cultures. In this approach the scrapings from the normal ovary and fallopian tube (fimbriated end) were collected during the surgery using an endoscopic kittner (e.g., Aspen Surgical) from patients undergoing surgery for benign gynecologic conditions. Among these patients we were able to establish cells in culture from the fallopian tube fimbria and the ovarian surface epithelium in approximately 75% and 30%, respectively, of the cases.

**Establishment of Cell Lines**

During the optimization period, we examined various medium formulations and cell collection methods which were tested on a total of 37 samples, including 18 tissue fragments that were collected at the pathology suite following surgery and 19 tissue scrapes that were collected in the operating room. The majority of the medium optimization had to be carried out on unmatched fallopian tube and ovarian samples because disease-free pairs of normal ovarian surface and fallopian tube epithelial cells are rarely available from the same patient, either because only one of the tissues was removed surgically, or one of the organs was involved with disease or was removed in a previous surgery. In addition, during the initial optimization period one of the cell pairs was sometimes lost due to growth arrest or cell death.

Once the conditions were optimized we established paired ovarian surface and fallopian tube epithelial cell lines from two patients who were 56 and 65 years old and did not have any type of gynecologic cancer. The ovaries and fallopian tubes from these patients were disease free. The tissue fragments that were collected after surgery were dissociated mechanically or enzymatically and plated in various formulations of WIT-fo medium. With this approach we were not able to establish any short term ovarian cells in culture, and only three fallopian tube cultures could be established.

The most successful method for cell culture establishment was to immediately place the ovarian and fallopian tube cells in WIT-fo cell culture media and transfer the cells to a tissue culture flask with a modified surface treatment (Primaria, BD Biosciences, Bedford, MA) and incubate at 37°C with 5% CO_2_ in ambient air. We strongly recommend the use of Primaria culture plates since it was nearly impossible to grow these cells using standard tissue culture plastic ware. Incubating the cells in lower O_2_ levels did not improve the results. WIT-fo is a modified version of WIT medium that we previously described [1] (Stemgent, Cambridge, MA). To prepare WIT-fo medium, the WIT medium was supplemented with EGF (0.01 ug/mL, E9644, Sigma-Aldrich, St. Louis, MO), Insulin (20 ug/mL, I0516, Sigma-Aldrich), Hydrocortisone (0.5 ug/mL, H0888, Sigma-Aldrich), 25ng/mL Cholera Toxin (227035, Calbiochem, EMD Millipore, Billerica, MA) and low concentrations (0.5 - 1%) of heat inactivated fetal bovine serum (HyClone, Thermo Fisher Scientific, Waltham, MA).

After the cells were in culture for 10-15 days, during which the medium was changed every 2-3 days, cells were lifted from the tissue culture plastic ware using 0.05% trypsin at room temperature while continuously checking and tapping the tissue culture flask to dislodge cells and therefore minimize exposure to trypsin (~15-30 seconds maximum exposure to trypsin or longer times only if necessary). Trypsin was inactivated using medium containing 10% serum, followed by centrifugation of cells in polypropylene tubes (500×g, 4 minutes) to remove excess trypsin and serum. Subcultures were established by seeding cells at a minimum density of 1×10^4^/cm^2^ (a split ratio of 1:2 was generally applied, i.e. one flask of cells was split and seeded into two equivalent sized flasks). We recommend counting cells to seed at the required minimum density rather than relying on a split ratio. Medium was replaced 24 hrs after re-plating cells and every 48-72 hours thereafter. Primary cell cultures were generally split every 1-2 weeks or when cells reached ~90% density.

The normal ovarian surface and fallopian tube epithelial cells were cultured in WIT-fo medium beyond 10 population doublings (Fig. 1A and B), while replicate plates of the same cells cultured under standard media conditions stopped growing after a few passages. We reported cell growth in terms of population doublings, or the log(2) of the number of cells harvested less the number of cells seeded. Hence 2 cells expand to 1,024 cells in 10 population doublings (2^10^= 1,024) and 10 population doublings is approximately equal to 3 orders of magnitude (×10^3^) net increase in cell numbers.

To culture ovarian epithelial cells, we tested several previously described culture media [3,4,5], including a 1:1 mixture of MCDB 105/Medium 199 with a range of 5-10% fetal bovine serum, 2 mM l-glutamine with and without 10 ng/ml epidermal growth factor, and Dulbecco’s modified Eagle’s medium (DMEM)/Ham's F-12 (1:1 mixture) with 10-15% fetal bovine serum. In neither case were we able to propagate ovarian epithelial cells beyond a few population doublings (data not shown). For fallopian tube epithelial culture we tested several previously described media [6,7,8], a 1:1 mixture of DMEM/Ham’s F12, supplemented with 0.1% BSA, 5% serum (1:1 mix of 2.5% fetal bovine serum plus 2.5 % Nu Serum (BD Biosciences, San Jose, CA)) and 17β estradiol, or a slightly modified version of this medium supplemented with 2% serum substitute. None of the above-mentioned traditional cell culture media that we tested supported long-term propagation of normal epithelial cells from human ovary or fallopian tube.

**Additional notes on culturing primary normal human fallopian tube and ovarian epithelial cells**

- Maintain the cells in large media volumes, which are greater than typical volumes;

e.g. T25 flask = 10mls; T75 flask = 28mls

6cm plate = 4mls; 10cm plate = 15mls

- Change media every 2-3 days, or sooner if the media turns a yellowish/brown color.
- **Trypsinization:** The OCE and FNE cells trypsinize quickly (<1 min when adding trypsin at room temperature); inactivate trypsin as soon as cells come off the flask, otherwise cells will not survive. Trypsinize using freshly defrosted 0.05% trypsin, followed by trypsin inactivation in 10-20% serum containing media (aliquot trypsin & freeze for this purpose). Alternatively, ‘Tryple Express’ (BD Biosciences) (designed for serum-free cell cultures) can be used to detach the cells from the plate (following the manufacturer’s instructions).
- **Cell seeding density:** Minimum seeding density ≥10,000 cells per cm^2^ of growth area (tissue culture plate), e.g. seed 400,000 cells into one T25 flask.
- **Freezing cells:** We have successfully used ‘Bambanker’ media to freeze OCE and FNE cells (Lymphotec Inc, Wako Chemicals, Richmond, VA). Cells can also be frozen in 10% DMSO in media containing 20% serum, but the recovery of viable cells has not been as optimal using this method.

**Retroviral infections**

Amphotropic retroviruses were produced by transfection of the 293T producer cell line with 1 µg of retroviral vector and 1 μg total of the packaging (pUMVC3) and envelope (pCMV-VSV-G) plasmids at an 8:1 ratio using Fugene 6 (Roche Applied Science, Indianapolis, IN). Viral supernatants were harvested at 24 and 48 hrs and used to infect primary ovarian surface and fallopian tube epithelial cells with 8 μg/ml polybrene. Retroviruses were sequentially introduced to recipient cells in individual steps in the following order: pmig-GFP-hTERT, pBABE-zeo-SV40-ER and pBABE-puro-H-ras V12. Selection of infected cells was performed serially and drug selection (500 μg/ml zeocin (zeo) and 1 μg/ml puromycin (puro)) was used to purify polyclonal infected populations after each infection. Primary ovarian surface epithelial cells were immortalized with hTERT between passages 2 to 6 and transformed between passages 26 to 30. Primary fallopian tube epithelial cells were transduced with hTERT between passages 1 to 4 and transformed at passage 16. Cells immortalized with hTERT and those that were transduced with SV40 and/ or H-ras were cultured in WIT-fo media on Primaria tissue culture ware (BD Biosciences). All protocols involving the use of retroviruses were approved by the Committee on Microbiological Safety.

Immortalized ovarian surface and fallopian tube epithelial cell lines (containing only the pmig-GFP-hTERT vector) are referred to as OCE and FNE and fully transformed derivatives as OCLER and FNLER following the introduction of vectors encoding hTERT (E), SV40 early region (L) and HRas (R). The FNE, OCE, FNLER and OCLER cells described in this manuscript are available from the Ince laboratory upon request.

**Analysis of tumorigenicity and metastasis**

The protocol for tumorigenesis experiments in immunocompromised mice was approved by the Harvard Standing Committee on Animals. All experiments were performed in compliance with relevant institutional and national guidelines and regulations. Single-cell suspensions were prepared in a Matrigel: WIT-fo mixture (1:1) and 1 million cells per 100 µl volume were injected in one intraperitoneal (IP) and two subcutaneous (SC) sites per mouse. Tumor cell injections were performed on 6-8 week old female immunodeficient nude (Nu/Nu) mice (Charles River Laboratories International, Inc, Wilmington, MA). Tumors were harvested 5 to 9 weeks after implantation of tumorigenic cells from tissue culture into IP and SC sites in nude mice. Tumor histopathology was assessed from hematoxylin and eosin stained sections from formalin-fixed paraffin-embedded (FFPE) tissues. Immunohistochemistry was carried out on FFPE tissues using cell type specific markers (CK7, FOXJ1, PAX8) to determine immunostaining patterns in mouse OCLER and FTLER xenografts as well as normal human ovaries and fallopian tubes (discarded tissues collected under an IRB-approved protocol) (see ‘Antibodies and experimental conditions’ table below for full details of immunostaining procedures). Metastasis of GFP-expressing tumor cells to lungs was analyzed initially using an Olympus SZX16 Stereo Fluorescence microscope in fresh tissues, followed by microscopic examination of hematoxylin and eosin stained sections of FFPE tissues. The presence of tumor cells in mouse lungs was confirmed by immunostaining for SV40 LT (v-300) and p53 (FL-393) (Santa Cruz Biotechnology, Santa Cruz, CA). Immunostaining was carried out using the conventional ABC technique (see ‘Antibodies and experimental conditions’ table below).

**Microarray data normalization and analysis**

Affymetrix microarrays of hTERT immortalized cell lines (OCE, FNE) (GEO Series accession number GSE37648) and publically available ovarian cancer datasets by Wu *et al*. [9] (GSE6008) and Tothill *et al*. [10] (GSE9891) were independently normalized using the variance stabilization method [11] (vsnrma) in R. Measurements of gene expression were assessed using 12 Human Genome U133 Plus 2 microarrays (HG U133Plus 2.0, Affymetrix, Santa Clara, CA) measuring 54,675 probesets. Samples that were arrayed included two biological replicates (paired hTERT immortalized OCE and FNE cells from two patients) and three experimental replicates (different passages) from each cell line. The R code and data used for this analysis are available at http://compbio.dfci.harvard.edu/pubs/OCFNLER/OCFNLER.zip.

To identify genes that were differentially expressed between paired hTERT immortalized FNE *vs*. OCE, we applied a modified t-test (False Discovery Rate (FDR) [12] adjusted *P*<0.05) using the duplicate correlation function in Limma [13] to block for patient differences. Setting the *P*-value < 0.05, 1,157 probesets were identified and included 632 probesets that were up-regulated in FNE *vs.* OCE and 525 probesets up-regulated in OCE *vs.* FNE (Supplementary Tables S1 and S2).

To classify human ovarian tumors from two publically available ovarian cancer datasets (detailed above) as fallopian tube (FT)-like and ovary (OV)-like, we sorted the FNE *vs* OCE genelist based on FDR-adjusted *P*-values and selected ten probesets with unique gene symbols that were over-expressed in either FNE or OCE. We then calculated the sum of the normalized expression values of these ten probesets in two ovarian cancer datasets by weighting FNE probesets by (+1) and OCE probesets by (-1). Specifically, the sum of the normalized expression values of OCE genes were subtracted from the sum of expression values of FNE genes to calculate a score for each tumor (e.g. a higher score tumor is more FT-like). We then fit a bimodal distribution of Gaussian curves using mixture modeling [14] to this score to identify two groups of tumors within each dataset, those that were more similar to normal ovarian epithelium (OV-like) or more similar to normal fallopian tube epithelium (FT-like).

We first performed this clustering in the Wu *et al.* [9] dataset that contains expression profiles of 99 fresh frozen, microdissected epithelial ovarian cancers (including many non-serous histologic subtypes) arrayed on a similar platform (Affymetrix HG U133A). Eight of the 10 selected probesets were available for analysis due to array platform differences. We used this cell-of-origin signature to define FT-like and OV-like subpopulations in the Wu data (using the methods described above) and visualized the distribution of these scores using density plots (Supplementary Fig. S4A). We evaluated the clinical characteristics of patient tumors classified as FT-like/OV-like and calculated their associated *P*-values using ordinal logistic regression (grade, stage) or Fisher’s Exact Test (histologic subtype).

The cell-of-origin signature was further validated in the Tothill *et al*. [10] dataset which included 246 serous and 20 endometrioid fresh frozen malignant tumors (not microdissected) that were arrayed on the HG U133 Plus 2.0 platform (Affymetrix) and gene expression profiles could be linked to patient survival data. Similar methods for Gaussian mixture modeling and tumor classification were used. To assess whether the FT-like/OV-like classification was associated with differences in patient disease-free and overall survival, we constructed Kaplan-Meier plots and calculated univariate *P*-values using the log-rank test. We also used a Cox proportional hazards test, adjusting for tumor grade and stage, serous histologic subtype, patient age and residual disease. All microarray and survival analyses were conducted using R

version 2.10.1.

**Antibodies and experimental conditions used in immunohistochemistry, immunofluorescence and western blotting**

| **Immunohistochemistry** | | |  |  | |  |  |  |
| --- | --- | --- | --- | --- | --- | --- | --- | --- |
| **Antigen** | **Manufacturer** | | **Catalog #** | **Host** | | **Antigen retrieval** | **Dilution** | **Incubation time** |
| Cytokeratin 7 | Abcam | | Ab68459 | Rabbit monoclonal | | Citrate PC | 1/100 | 1hr (Envision) |
| FOXJ1 | Sigma-Aldrich | | HPA005714 | Rabbit polyclonal | | Citrate PC | 1/250 | 1hr (Envision) |
| PAX8 | ProteinTech | | 10336-1-AP | Rabbit polyclonal | | Citrate PC | 1/800 | 1hr (Envision) |
| SV40 (v-300) | Santa Cruz | | sc-20800 | Rabbit polyclonal | | Citrate PC | 1/100 | 1hr (Envision) |
| p53 (FL-393) | Santa Cruz | | sc-6243 | Rabbit polyclonal | | Citrate PC | 1/100 | 1hr (Envision) |
| **Immunofluorescence** | | |  |  | |  |  |  |
| **Antigen** | **Manufacturer** | | **Catalog #** | **Host** | | **Antigen retrieval** | **Dilution** | **Incubation time** |
| PAX8 | ProteinTech | | 10336-1-AP | Rabbit polyclonal | | 2-4% PFM fixation, permeabilization with 0.2-0.25% Triton-X 10-15m at 4ºC | 1:150 | 2hr 4ºC |
| FOXJ1 | Sigma-Aldrich | | HPA005714 | Rabbit polyclonal | | same as above | 1:150 | 2hr 4ºC |
| **Western Blotting** | | | |  |  |  |  |  |
| **Antigen** | | **Manufacturer** | | **Catalog #** | **Host** | **Dilution** | **Incubation time** | **Temperature** |
| β-Actin (AC-15) | | Sigma-Aldrich | | A1978 | Mouse monoclonal | 1/5000 | 1h | RT |
| Cytokeratin 7 (OV-TL 12/30) | | Millepore | | MAB3554 | Mouse monoclonal | 1/2000 | O’N | 4ºC |
| FOXJ1 | | Sigma-Aldrich | | HPA005714 | Rabbit polyclonal | 1/500 | 1h | RT |
| PAX8 | | ProteinTech Group | | 10336-1-AP | Rabbit polyclonal | 1/1000 | O’N | 4ºC |

(Supplementary Tables S1 & S2 are provided as separate files)

**Supplementary Table S3**. Total tumor burden and time of tumor incubation in the OCLER/FNLER xenograft model where lung metastases were evaluated ^a^.

| **Cell line** | **Genotype** | **Total tumor burden** ^b^ **per mouse** (g) ± s.d. | **Tumor incubation**  (wks) |
| --- | --- | --- | --- |
| OCLER | *hTERT*, SV40 *ER*, *HRAS^V12^* | 3.90 ± 1.31 | 6.50 ± 1.31 |
| FNLER | *hTERT*, SV40 *ER*, *HRAS^V12^* | 4.94 ± 2.36 | 7.33 ± 1.37 |
|  |  | *P* ^c^ = 0.70 | *P* ^c^ = 0.28 |

^a^ Lungs from eight mice with OCLER tumors and six mice with FNLER tumors were examined. Five total mice with OCLER tumors were excluded from this analysis because their lungs were not collected (n=3) or their total tumor burden was <0.5g (n=2).

^b^ Values are means ± s.d. across all mice injected with each cell type that had a total tumor burden ≥0.5g per mouse (the sum of 1× intraperitoneal and 2× subcutaneous sites per mouse).

^c^ *P*-values were calculated using the Mann-Whitney non-parametric test.

(Supplementary Figures S1 & S2 are provided as separate files)

**Supplementary Fig. S3.** Growth of hTERT-immortalized FNE and OCE cells in WIT-fo versus unmodified WIT medium.

| 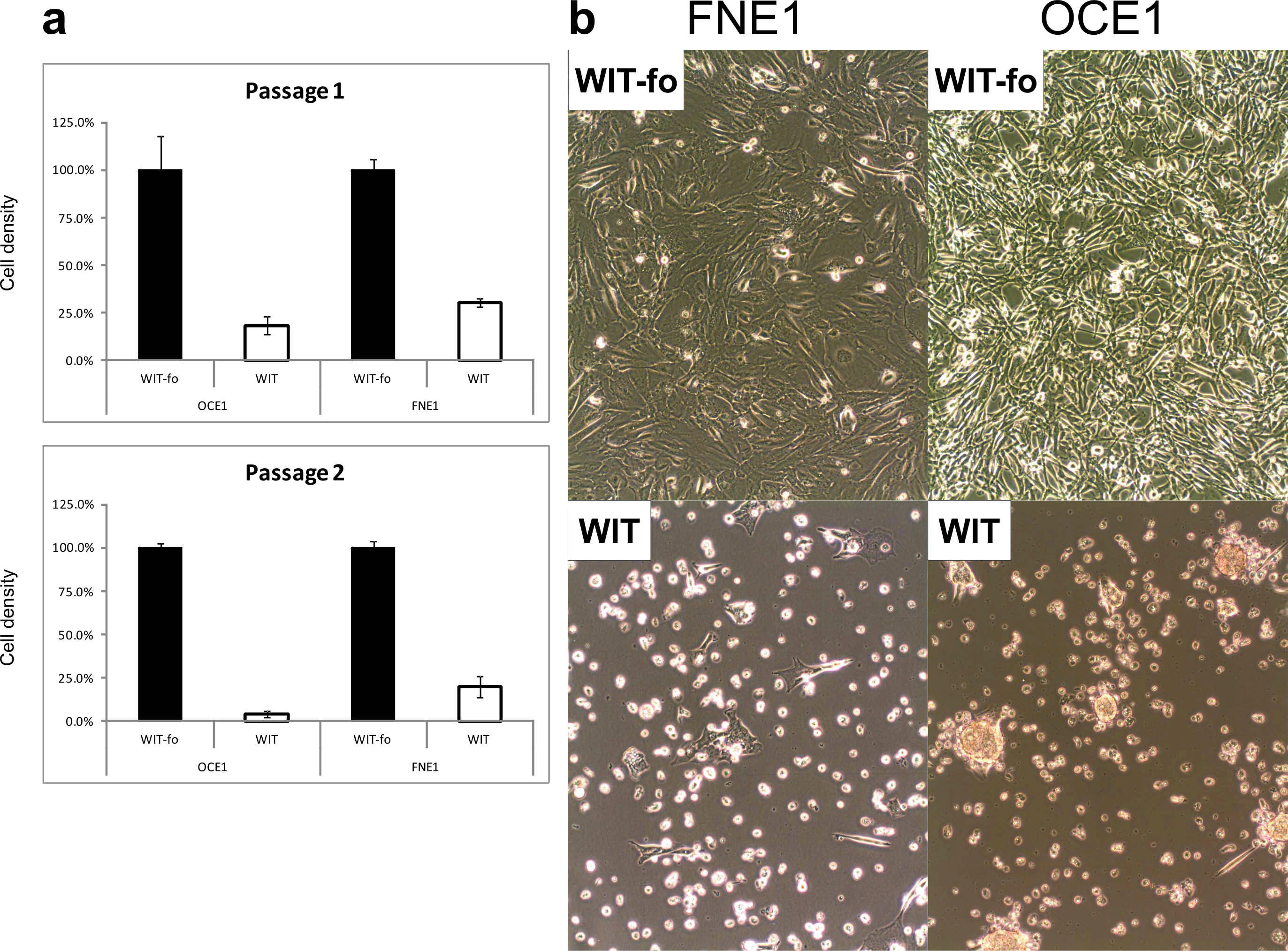 |
| --- |

**A,** Replicate plates of OCE1 and FNE1 cells using WIT-fo or WIT media demonstrates that cell viability rapidly decreases in the WIT media. OCE1/FNE1 indicates that these hTERT immortalized cells were from patient 1.

**B,** Pictured OCE1/FNE1 cells grow to confluence in WIT-fo media as compared with widespread cell death in replicate plates grown in WIT media (pictured cells represent those that were counted at passage 2 (panel a)).

**Supplementary Fig. S4A.** Hierarchical clustering and principal components analyses based on global gene expression profiles of normal human fallopian tube epithelium and ovarian surface epithelium assessed using the HG U133 Plus 2.0 (Affymetrix) microarrays in four previously published datasets (GEO GSE14407, GSE28044, GSE19383, GSE29450). The vsnrma normalization was used to normalize the combined dataset. (See figure legends on next page).

| 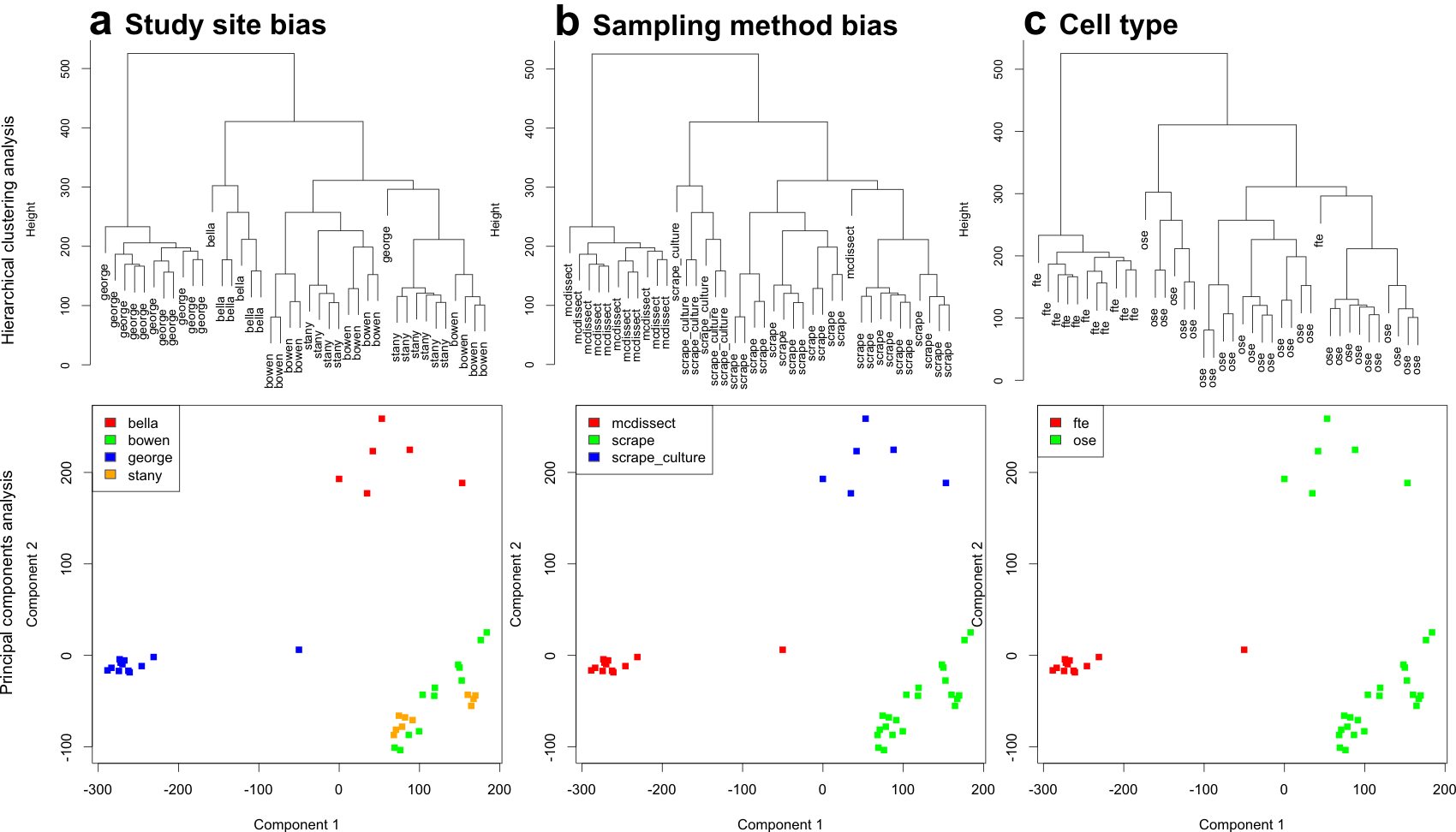 |
| --- |

**Supplementary Fig. S4A figure legends**

Four previously published datasets were available that had profiled either normal human ovarian surface epithelium or fallopian tube epithelium using HG U133 Plus 2.0 (Affymetrix) arrays [15,16,17,18]. In the top panes, dendrograms are presented from complete linkage hierarchical clustering (euclidean distance) based on global gene expression profiles. In the bottom panes, results from principal components analysis also based on global gene expression profiles are presented. Columns a, b and c demonstrate the results from the same analyses using different labels to indicate:

**A**, the **study** that the samples were from (George *et al*., 2011; Bellacosa *et al*., 2010 (bella); Bowen *et al*., 2009; Stany *et al*., 2011),

**B**, the **sample collection method** (fallopian tube epithelium microdissected from a specimen (mcdissect), ovarian surface epithelial scraping (scrape), or cultured ovarian surface epithelial scraping (scrape_culture)),

**C**, the **cell type**- ovarian surface epithelium (OSE) or fallopian tube epithelium (FTE).

These data highlight the value of evaluating matched pairs of ovarian epithelial and fallopian tube epithelial cells that are isolated from the same patients and cultured/treated under identical conditions.

In previous studies a single cell type was analyzed; thus the normal ovarian epithelial and fallopian tube epithelial cells were profiled in separate studies, sampled using different methods, and the ovarian and fallopian tube epithelium were not patient matched. As a result the samples that are from different studies formed separate clusters in the principal component analysis. This result suggests that some of the apparent differences between unmatched fallopian tube vs. ovarian epithelial cell comparisons may be due to patient-to-patient variation, technical variation due to study site or differences in collection methods and may not reflect true cellular differences.

**Supplementary Fig. S4B.** Validation of the ten probeset cell-of-origin signature in four publically available datasets (GEO GSE14407, GSE28044, GSE19383, GSE29450) that had profiled normal human fallopian tube epithelium and ovarian surface epithelium using the same HG U133 Plus 2.0 (Affymetrix) microarray platform.

| 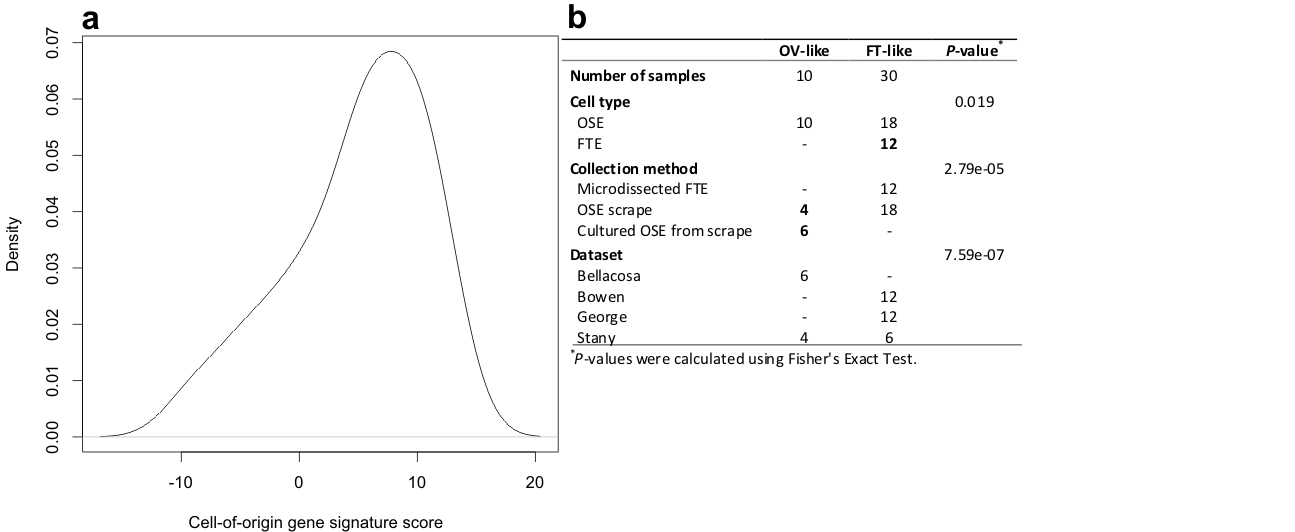 |
| --- |

**A**, Density plot of the ten probeset FNE *vs*. OCE cell-of-origin signature score shows a left-skewed distribution of OV/FT-like scores in normal ovarian surface epithelium and normal fallopian tube epithelium samples from four independent datasets.

**B**, The application of two Gaussian curves to classify samples as normal OV-like or normal FT-like using the FNE *vs*. OCE signature correctly classified 12/12 microdissected FTE samples as FT-like. Furthermore, 6/6 of the ovarian surface epithelial scrapes that had been cultured were classified as OV-like. The signature did not discriminate uncultured ovarian surface epithelial scrapes as well, although 4/22 were classified as OV-like while the remaining 18 were classified as FT-like. This may be due to the cellular heterogeneity and the presence of stromal cells in ovarian scrapes that could contribute to the gene expression profile in these samples.

**Supplementary Fig. S5.** Comparisons of the OCE *vs*. FNE cell-of-origin gene signature as compared with 10-probeset signatures generated by random permutation in the Wu *et al*. [9] dataset.

| **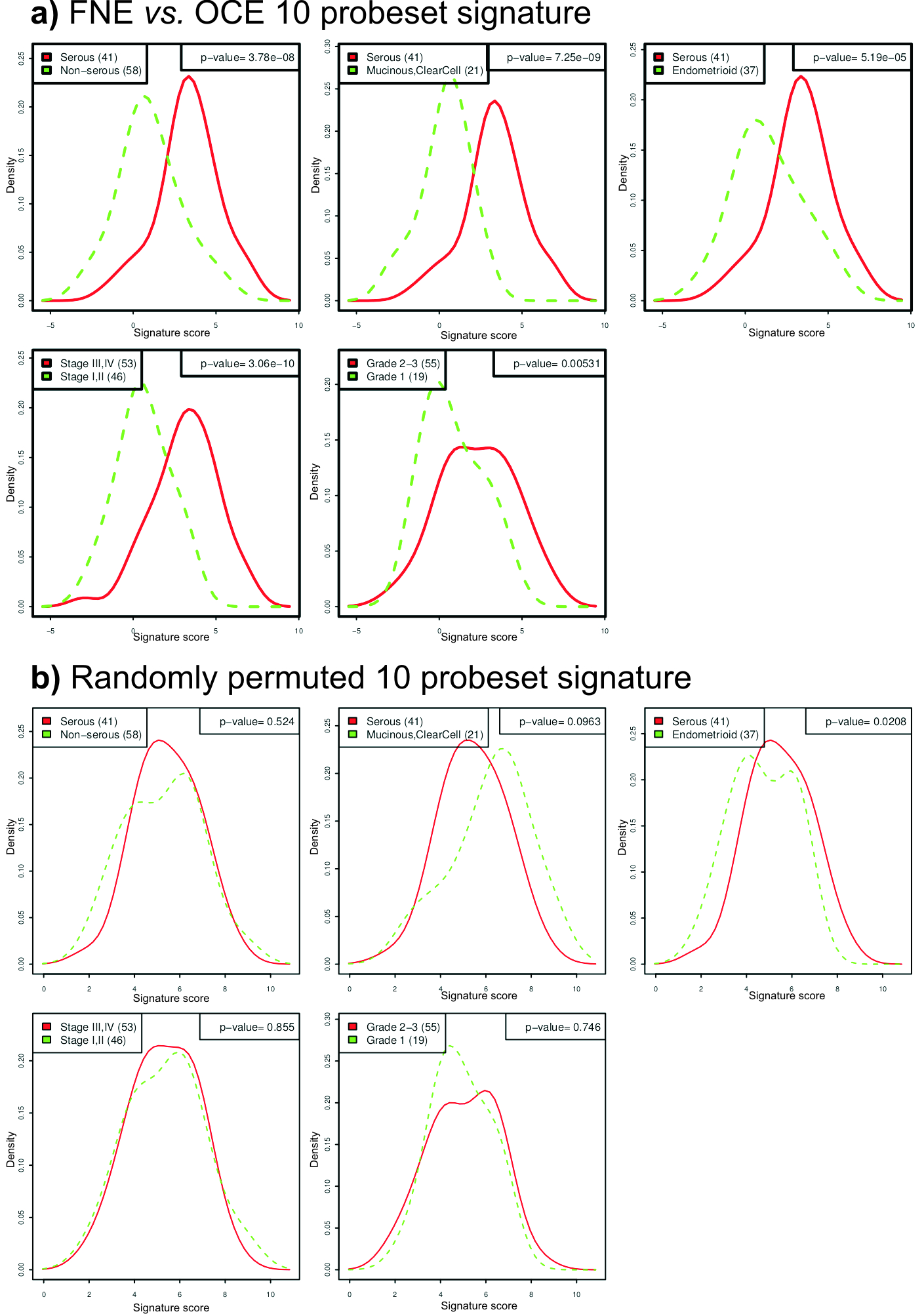** |
| --- |

**A**, Density plots demonstrate that the continuous distribution of the cell-of-origin signature scores differ between tumors grouped by histological subtype, tumor stage and grade (*P*-value (Welch two-sample t-test) ≤ 0.005).

**B**, Density plots show the continuous distribution of a randomly permuted 10 probeset gene signature score which demonstrates that the random signature is not associated with any tumor characteristics.

**Supplementary Figure S6.** FNLER derived tumors in an immunocompromised mouse xenograft model demonstrate increased potential for lung metastasis.

| 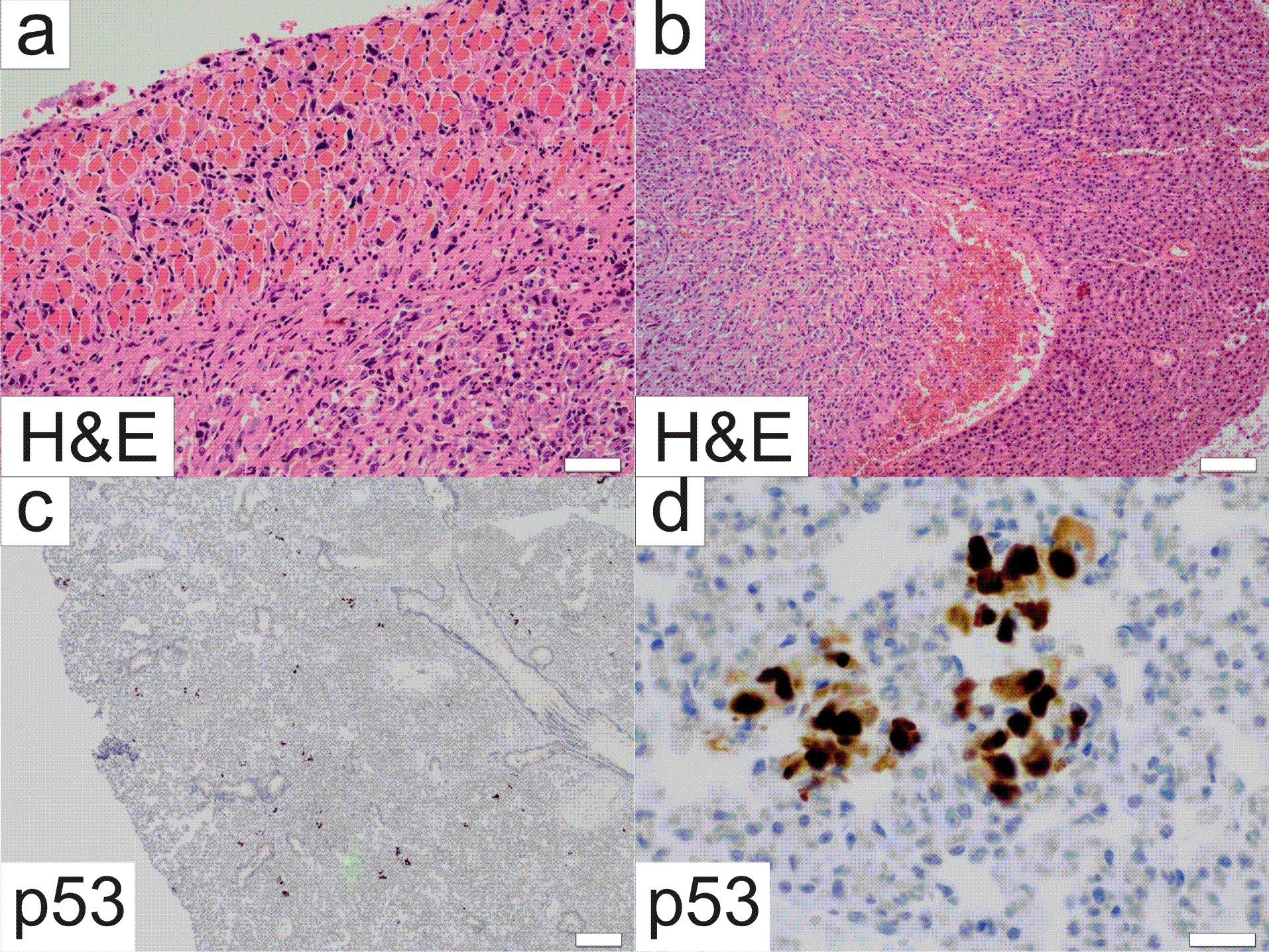 |
| --- |

(**a-b**) FNLER derived tumors were observed infiltrating into the surrounding intraperitoneal tissues including diaphragm (**a**) and liver (**b**). These highly invasive tumors caused death due to tumor in the mice (scale bars = 50µM and 100µM, respectively).

**(c-d**) Metastatic FNLER cells were confirmed in mouse lung FFPE sections as positive staining for p53 (scale bars = 200µM and 20 µM, respectively).

**Supplementary Figure S7.** Evaluation of metastases to mouse lungs from intraperitoneal/subcutaneous FNLER or OCLER tumors.


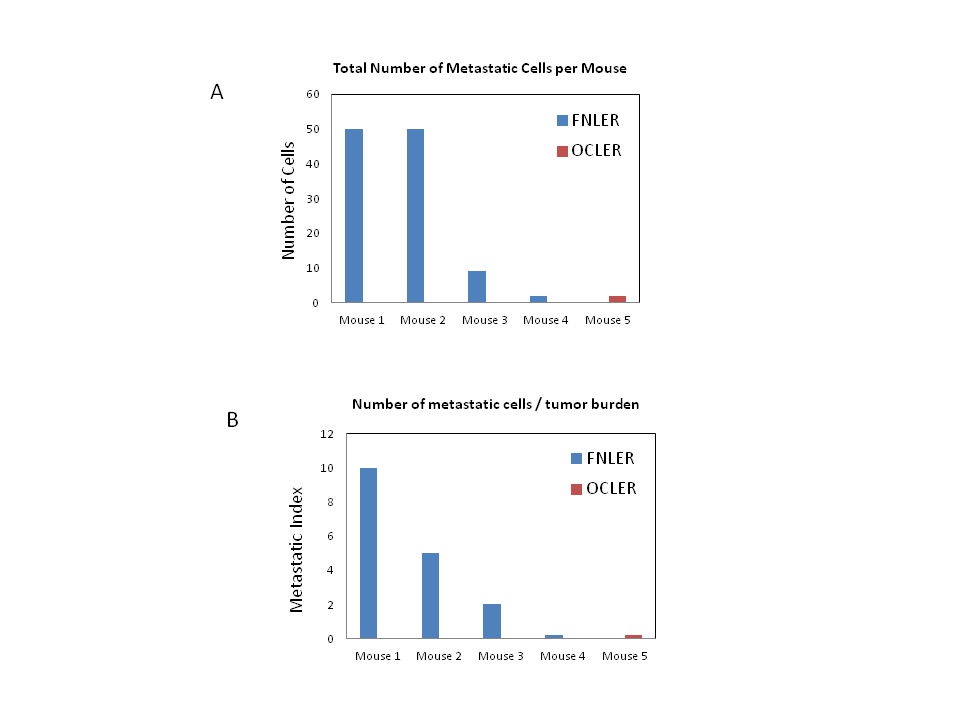


**A**, We detected lung metastases in 4 mice with FNLER xenograft tumors and 1 mouse with OCLER tumors. The total number of metastatic cells to mouse lungs from intraperitoneal/subcutaneous tumors was higher in mice bearing FNLER-derived tumors (mouse 1 = 50 cells; mouse 2 = 50 cells; mouse 3 = 9 cells; mouse 4 = 2 cells) compared to the single OCLER xenograft with 2 metastatic cells in the lung.

**B**, Each bar represents the metastatic index calculated by dividing the number of metastatic cells by the total tumor burden [g] for each mouse.

**References**

1. Ince TA, Richardson AL, Bell GW, Saitoh M, Godar S, et al. (2007) Transformation of Different Human Breast Epithelial Cell Types Leads to Distinct Tumor Phenotypes. Cancer Cell 12: 160-170.

2. Brenneman M, Rigby P (1968) Protein electrophoretic patterns of serum and peritoneal fluid in normal, tumor-bearing, and immune mice. Cancer Res 28: 1138-1142.

3. Auersperg N, Maines-Bandiera SL, Dyck HG (1997) Ovarian carcinogenesis and the biology of ovarian surface epithelium. J Cell Physiol 173: 261-265.

4. Auersperg N, Maines-Bandiera SL, Dyck HG, Kruk PA (1994) Characterization of cultured human ovarian surface epithelial cells: phenotypic plasticity and premalignant changes. Lab Invest 71: 510-518.

5. Gregoire L, Munkarah A, Rabah R, Morris RT, Lancaster WD (1998) Organotypic culture of human ovarian surface epithelial cells: a potential model for ovarian carcinogenesis. In Vitro Cell Dev Biol Anim 34: 636-639.

6. Comer MT, Leese HJ, Southgate J (1998) Induction of a differentiated ciliated cell phenotype in primary cultures of Fallopian tube epithelium. Hum Reprod 13: 3114-3120.

7. Briton-Jones C, Lok IH, Yuen PM, Chiu TT, Cheung LP, et al. (2002) Human oviductin mRNA expression is not maintained in oviduct mucosal cell culture. Fertil Steril 77: 576-580.

8. Levanon K, Ng V, Piao HY, Zhang Y, Chang MC, et al. (2010) Primary ex vivo cultures of human fallopian tube epithelium as a model for serous ovarian carcinogenesis. Oncogene 29: 1103-1113.

9. Wu R, Hendrix-Lucas N, Kuick R, Zhai Y, Schwartz DR, et al. (2007) Mouse model of human ovarian endometrioid adenocarcinoma based on somatic defects in the Wnt/beta-catenin and PI3K/Pten signaling pathways. Cancer Cell 11: 321-333.

10. Tothill RW, Tinker AV, George J, Brown R, Fox SB, et al. (2008) Novel molecular subtypes of serous and endometrioid ovarian cancer linked to clinical outcome. Clin Cancer Res 14: 5198-5208.

11. Huber W, von Heydebreck A, Sultmann H, Poustka A, Vingron M (2002) Variance stabilization applied to microarray data calibration and to the quantification of differential expression. Bioinformatics 18 Suppl 1: S96-104.

12. Hochberg Y, Benjamini Y (1990) More powerful procedures for multiple significance testing. Stat Med 9: 811-818.

13. Smyth GK (2004) Linear models and empirical bayes methods for assessing differential expression in microarray experiments. Stat Appl Genet Mol Biol 3: Article3.

14. Fraley C, Raftery AE (2006) MCLUST Version 3 for R: Normal Mixture Modeling and Model-Based Clustering.

15. Bellacosa A, Godwin AK, Peri S, Devarajan K, Caretti E, et al. (2010) Altered gene expression in morphologically normal epithelial cells from heterozygous carriers of BRCA1 or BRCA2 mutations. Cancer Prev Res (Phila) 3: 48-61.

16. Bowen NJ, Walker LD, Matyunina LV, Logani S, Totten KA, et al. (2009) Gene expression profiling supports the hypothesis that human ovarian surface epithelia are multipotent and capable of serving as ovarian cancer initiating cells. BMC Med Genomics 2: 71.

17. George SH, Greenaway J, Milea A, Clary V, Shaw S, et al. (2011) Identification of abrogated pathways in fallopian tube epithelium from BRCA1 mutation carriers. J Pathol 225: 106-117.

18. Stany MP, Vathipadiekal V, Ozbun L, Stone RL, Mok SC, et al. (2011) Identification of novel therapeutic targets in microdissected clear cell ovarian cancers. PLoS One 6: e21121.
